# Supplementary material for: TOPOVIBL-REC114 interaction regulates meiotic DNA double-strand breaks
Source: Nat Commun. 2022 Nov 17;13:7048. doi: 10.1038/s41467-022-34799-0 (PMC9671922; doi:10.1038/s41467-022-34799-0)
Supplement: Supplementary file 1 — Supplementary Information [file 41467_2022_34799_MOESM1_ESM.pdf]

Supplementary data

Supplementary Figures 1-16

Supplementary Tables 1-4

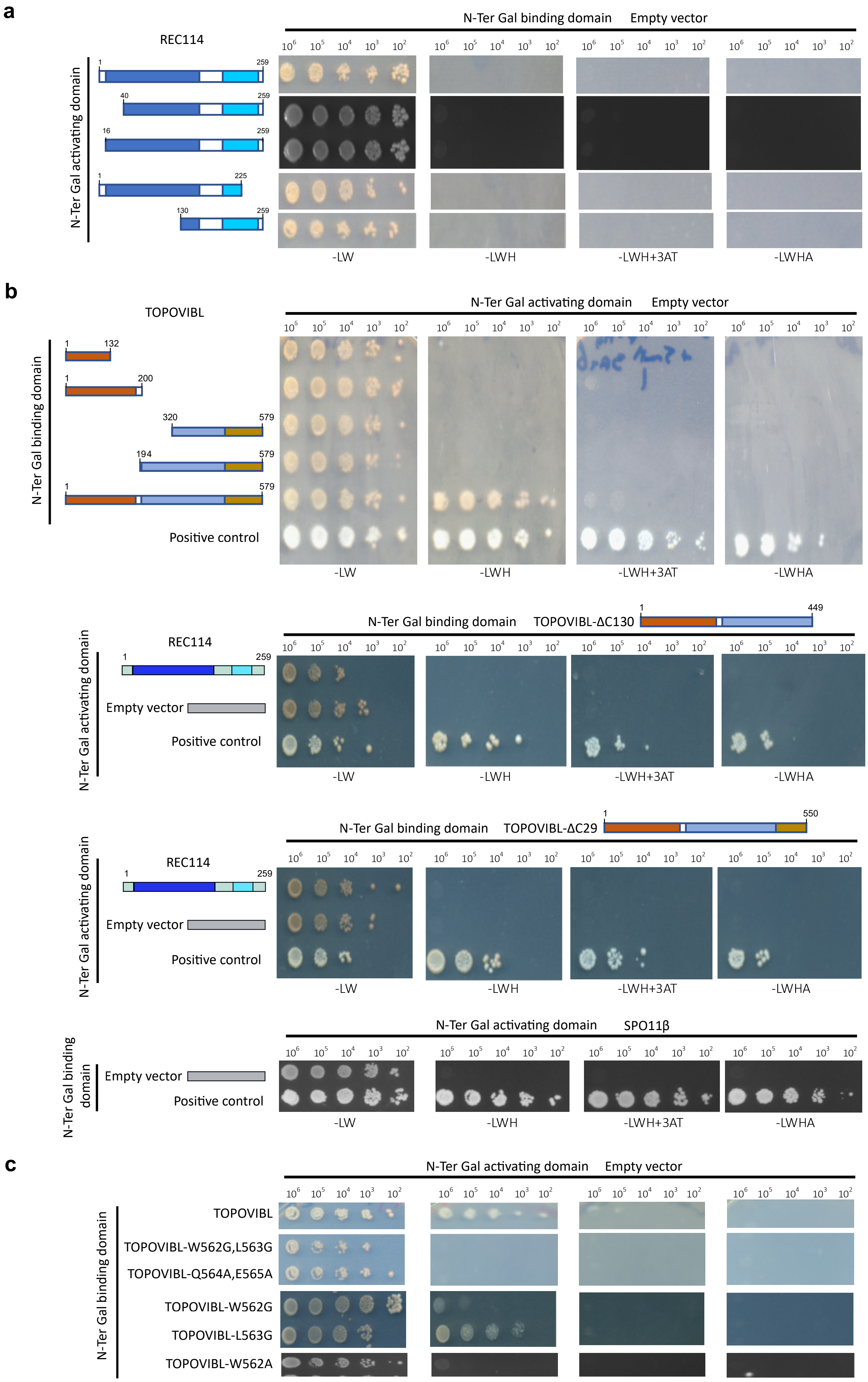
a
N-Ter Gal binding domain Empty vector
REC114
N-Ter Gal activating domain
1 259
40 259
16 259
1 225
130 259
10^6 10^5 10^4 10^3 10^2
-LW
-LWH
-LWH+3AT
-LWHA
b
N-Ter Gal activating domain Empty vector
TOPOVIBL
N-Ter Gal binding domain
1 132
1 200
320 579
194 579
1 579
Positive control
-LW
-LWH
-LWH+3AT
-LWHA
N-Ter Gal binding domain TOPOVIBL-ΔC130
1 449
REC114
N-Ter Gal activating domain
1 259
Empty vector
Positive control
-LW
-LWH
-LWH+3AT
-LWHA
N-Ter Gal binding domain TOPOVIBL-ΔC29
1 550
REC114
N-Ter Gal activating domain
1 259
Empty vector
Positive control
-LW
-LWH
-LWH+3AT
-LWHA
N-Ter Gal activating domain SPO11β
N-Ter Gal binding domain
Empty vector
Positive control
-LW
-LWH
-LWH+3AT
-LWHA
c
N-Ter Gal activating domain Empty vector
N-Ter Gal binding domain
TOPOVIBL
TOPOVIBL-W562G,L563G
TOPOVIBL-Q564A,E565A
TOPOVIBL-W562G
TOPOVIBL-L563G
TOPOVIBL-W562A

Supplementary Figure 1. Controls for Y2H

a. Negative controls for Y2H assays for Fig. 1b

b. Negative controls for Y2H assays for Fig. 1c

c. Negative controls for Y2H assays for Fig. 2h.

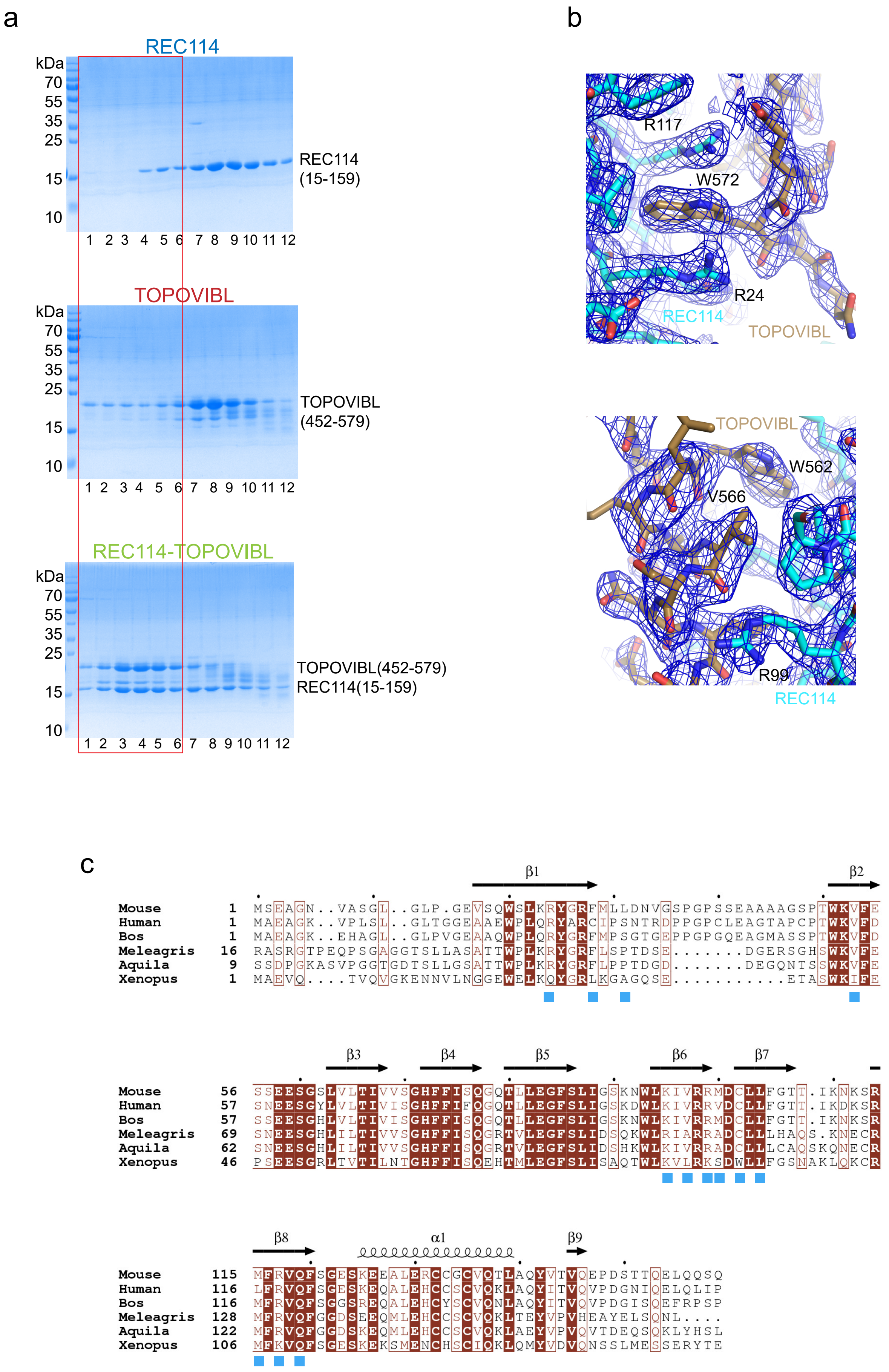
a
REC114
kDa
70
55
35
25
15
10
1 2 3 4 5 6 7 8 9 10 11 12
REC114
(15-159)
TOPOVIBL
TOPOVIBL
(452-579)
REC114-TOPOVIBL
TOPOVIBL(452-579)
REC114(15-159)
b
R117
W572
R24
REC114
TOPOVIBL
TOPOVIBL
W562
V566
R99
REC114
c
β1
β2
Mouse 1 MSEAGN..VASGL..GLP.GEVSQWSLKRYGRFMLLDNVGSPGPSSEAAAAGSPTWKVFE
Human 1 MAEAGK..VPLSL..GLTGGEAAEWPLQRYARCIPSNTRDPPGPCLEAGTAPCPTWKVFD
Bos 1 MAEAGK..EHAGL..GLPVGEAAQWPLQRYGRFMPSGTGEPPGPGQEAGMASSPTWKVFD
Meleagris 16 RASRGTPEQPSGAGGTSLLASATTWPLKRYGRFLSPTDSE.......DGERSGHSWKVFE
Aquila 9 SSDPGKASVPGGTGDTSLLGSATTWPLKRYGRFLPPTDGD.......DEGQNTSSWKVFE
Xenopus 1 MAEVQ....TVQVGKENNVLNGGEWELKQYGRLKGAGQSE...........ETASWKIFE
β3
β4
β5
β6
β7
Mouse 56 SSEESGSLVLTIVVSGHFFISQGQTLLEGFSLIGSKNWLKIVRRMDCLLFGTT.IKNKSR
Human 57 SNEESGYLVLTIVISGHFFIFQGQTLLEGFSLIGSKDWLKIVRRVDCLLFGTT.IKDKSR
Bos 57 SSEESGHLVLTIVISGHFFISQGQTLLEGFSLIGSKNWLKIVRRMDCLLFGTT.IKNKSR
Meleagris 69 SNEESGHLILTIVVSGHFFISQGRTVLEGFSLIDSQKWLRIARRADCLLLHAQS.KNECR
Aquila 62 SNEESGHLILTIVVSGHFFISQGRTVLEGFSLIDSHKWLKIVRRADCLLLCAQSKQNECR
Xenopus 46 PSEESGRLTVTILNTGHFFISQEHTMLEGFSLISAQTWLKVLRKSDWLLFGSNAKLQKCR
β8
α1
β9
Mouse 115 MFRVQFSGESKEEALERCCGCVQTLAQYVTVQEPDSTTQELQQSQ
Human 116 LFRVQFSGESKEQALEHCCSCVQKLAQYITVQVPDGNIQELQLIP
Bos 116 MFRVQFSGGSREQALEHCYSCVQNLAQYITVQVPDGISQEFRPSP
Meleagris 128 MFRVQFGGDSEEQMLEHCCSCVQKLTEYVPVHEAYELSQNL....
Aquila 122 MFRVQFGGDSKEQMLEHCCSCVQKLAEYVPVQVTDEQSQKLYHSL
Xenopus 106 MFKVQFSGESKEKSMENCHSCIQKLQMYVDVQNSSLMESSERYTE

Supplementary Figure 2. Properties of the REC114-TOPOVIBL complex

a. SDS-PAGE analysis of fractions 1-12 of the Superdex 200 gel filtration elution profiles of REC114$^{15-159}$, TOPOVIBL$^{452-579}$ and their complex shown in Fig. 1e. The red rectangle highlights fractions containing the REC114-TOPOVIBL complex. Source data are provided as a Source Data file.

b. Representative part of the 2*F*o-*F*c electron density map covering the TOPOVIBL-REC114 interface contoured at 1.0 σ.

c. Sequence alignment of the REC114 PH domain from *M. musculus*, *H. sapiens*, *B. mutus*, *Meleagris gallopavo*, *Aquila chrisaetos canadensis* and *Xenopus laevis* used to highlight the amino-acid conservation on the structure (Fig. 2c). Brown letters: equivalent amino acids; white letters: identical amino acids. The residues interacting with TOPOVIBL are shown as blue squares.

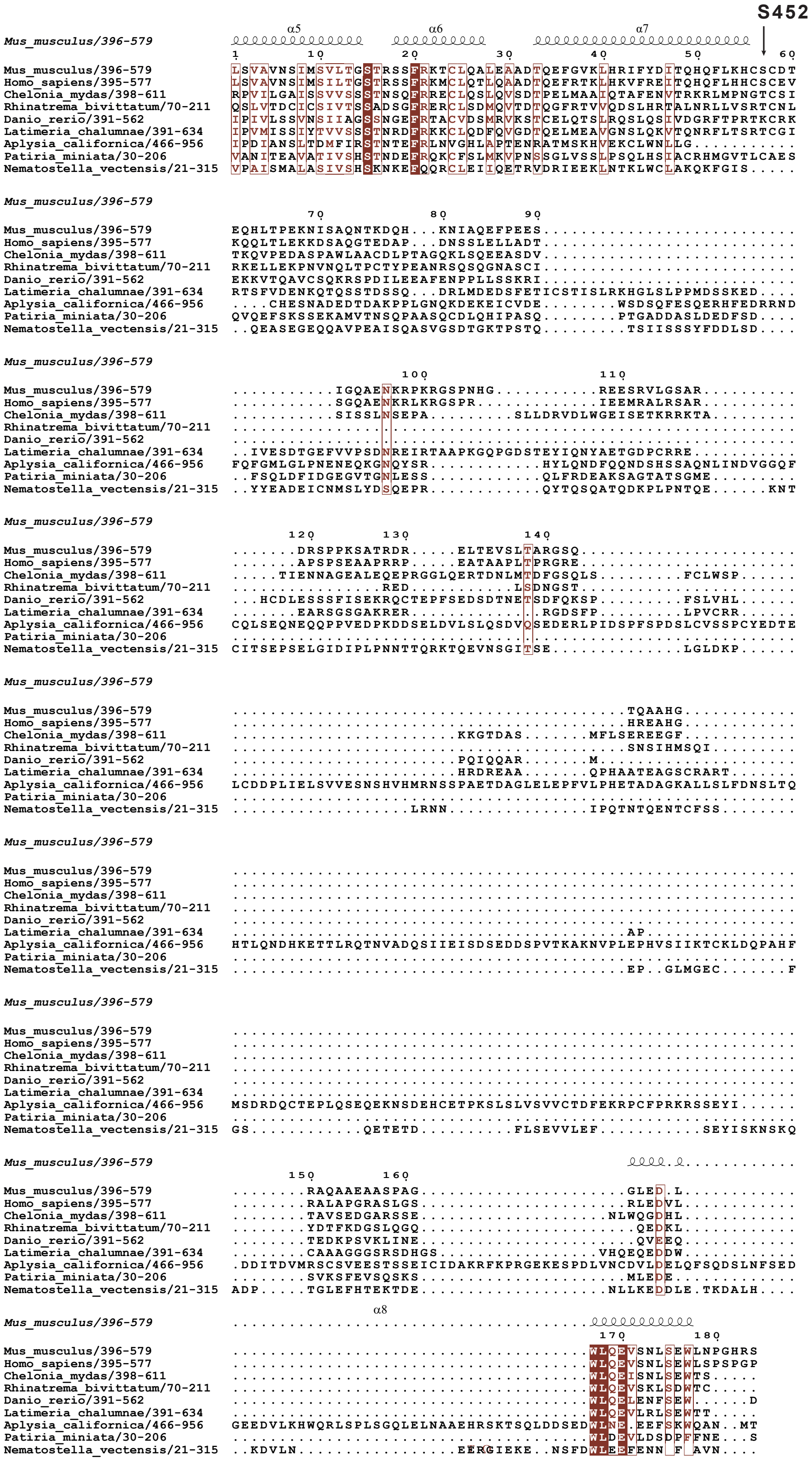

S452
α5 α6 α7
Mus_musculus/396-579
1 10 20 30 40 50 60
Mus_musculus/396-579 LSVAVNSIMSVLTGSTRSSFRKTCLQALEAADTQEFGVKLHRIFYDITQHQFLKHCSCDT
Homo_sapiens/395-577 LSVAVNSIMSILTGSTRSSFRKMCLQTLQAADTQEFRTKLHKVFREITQHQFLHHCSCEV
Chelonia_mydas/398-611 RPVILGAISSVVSSSTNSQFRRECLQSLQVSDTPELMAAIQTAFENVTRKRLMPNGTCSI
Rhinatrema_bivittatum/70-211 QSLVTDCICSIVTSSADSGFRERCLSDMQVTDTQGFRTVVQDSLHRTALNRLLVSRTCNL
Danio_rerio/391-562 IPIVLSSVNSIIAGSSNGEFRTACVDSMRVKSTCELQTSLRQSLQSIVDGRFTPRTKCRK
Latimeria_chalumnae/391-634 IPVMISSIYTVVSSSTNRDFRKKCLQDFQVGDTQELMEAVGNSLQKVTQNRFLTSRTCGI
Aplysia_californica/466-956 IPDIANSLTDMFIRSTNTEFRLNVGHLAPTENRATMSKHVEKCLWNLLG...........
Patiria_miniata/30-206 VANITEAVATIVSHSTNDEFRQKCFSLMKVPNSSGLVSSLPSQLHSIACRHMGVTLCAES
Nematostella_vectensis/21-315 VPAISMALASIVSHSKNKEFQQRCLEIIQETRVDRIEEKLNTKLWCLAKQKFGIS.....
Mus_musculus/396-579
70 80 90
Mus_musculus/396-579 EQHLTPEKNISAQNTKDQH...KNIAQEFPEES...........................
Homo_sapiens/395-577 KQQLTLEKKDSAQGTEDAP...DNSSLELLADT...........................
Chelonia_mydas/398-611 TKQVPEDASPAWLAACDLPTAGQKLSQEEASDV...........................
Rhinatrema_bivittatum/70-211 RKELLEKPNVNQLTPCTYPEANRSQSQGNASCI...........................
Danio_rerio/391-562 EKKVTQAVCSQKRSPDILEEAFENPPLLSSKRI...........................
Latimeria_chalumnae/391-634 RTSFVDENKQTQSSTDSSQ...DRLMDEDSFETICSTISLRKHGLSLPPMDSSKED....
Aplysia_californica/466-956 ....CHESNADEDTDAKPPLGNQKDEKEICVDE........WSDSQFESQERHFEDRRND
Patiria_miniata/30-206 QVQEFSKSSEKAMVTNSQPAASQCDLQHIPASQ........PTGADDASLDEDFSD....
Nematostella_vectensis/21-315 ..QEASEGEQQAVPEAISQASVGSDTGKTPSTQ.........TSIISSSYFDDLSD....
Mus_musculus/396-579
100 110
Mus_musculus/396-579 ...........IGQAENKRPKRGSPNHG...........REESRVLGSAR..........
Homo_sapiens/395-577 ...........SGQAENKRLKRGSPR.............IEEMRALRSAR..........
Chelonia_mydas/398-611 ...........SISSLNSEPA.........SLLDRVDLWGEISETKRRKTA.........
Rhinatrema_bivittatum/70-211 ....
Danio_rerio/391-562 ....
Latimeria_chalumnae/391-634 ..IVESDTGEFVVPSDNREIRTAAPKGQPGDSTEYIQNYAETGDPCRRE...........
Aplysia_californica/466-956 FQFGMLGLPNENEQKGNQYSR...........HYLQNDFQQNDSHSSAQNLINDVGGQF
Patiria_miniata/30-206 ..FSQLDFIDGEGVTGNLESS...........QLFRDEAKSAGTATSGME.........
Nematostella_vectensis/21-315 ..YYEADEICNMSLYDSQEPR...........QYTQSQATQDKPLPNTQE......KNT
Mus_musculus/396-579
120 130 140
Mus_musculus/396-579 .......DRSPPKSATRDR.....ELTEVSLTARGSQ.......................
Homo_sapiens/395-577 .......APSPSEAAPRRP.....EATAAPLTPRGRE.......................
Chelonia_mydas/398-611 .....TIENNAGEALEQEPRGGLQERTDNLMTDFGSQLS.........FCLWSP......
Rhinatrema_bivittatum/70-211 ................RED..........LSDNGST.......................
Danio_rerio/391-562 ...HCDLESSSFISEKRQCTEPFSEDSDTNETSDFQKSP.........FSLVHL......
Latimeria_chalumnae/391-634 .......EARSGSGAKRER............RGDSFP.........LPVCRR......
Aplysia_californica/466-956 CQLSEQNEQQPPVEDPKDDSELDVLSLQSDVQSEDERLPIDSPFSPDSLCVSSPCYEDTE
Patiria_miniata/30-206 ....
Nematostella_vectensis/21-315 CITSEPSELGIDIPLPNNTTQRKTQEVNSGITSE..............LGLDKP......
Mus_musculus/396-579
Mus_musculus/396-579 ....TQAAHG............
Homo_sapiens/395-577 ....HREAHG............
Chelonia_mydas/398-611 ........................KKGTDAS.......MFLSEREEGF............
Rhinatrema_bivittatum/70-211 ....SNSIHMSQI.........
Danio_rerio/391-562 ........................PQIQQAR.......M.....................
Latimeria_chalumnae/391-634 ........................HRDREAA.......QPHAATEAGSCRART.......
Aplysia_californica/466-956 LCDDPLIELSVVESNSHVHMRNSSPAETDAGLELEPFVLPHETADAGKALLSLFDNSLTQ
Patiria_miniata/30-206 ....
Nematostella_vectensis/21-315 ....................LRNN................IPQTNTQENTCFSS........
Mus_musculus/396-579
Mus_musculus/396-579 ....
Homo_sapiens/395-577 ....
Chelonia_mydas/398-611 ....
Rhinatrema_bivittatum/70-211 ....
Danio_rerio/391-562 ....
Latimeria_chalumnae/391-634 ....AP..............
Aplysia_californica/466-956 HTLQNDHKETTLRQTNVADQSIIEISDSEDDSPVTKAKNVPLEPHVSIIKTCKLDQPAHF
Patiria_miniata/30-206 ....
Nematostella_vectensis/21-315 ....EP..GLMGEC.......F
Mus_musculus/396-579
Mus_musculus/396-579 ....
Homo_sapiens/395-577 ....
Chelonia_mydas/398-611 ....
Rhinatrema_bivittatum/70-211 ....
Danio_rerio/391-562 ....
Latimeria_chalumnae/391-634 ....
Aplysia_californica/466-956 MSDRDQCTEPLQSEQEKNSDEHCETPKSLSLVSVVCTDFEKRPCFPRKRSSEYI......
Patiria_miniata/30-206 ....
Nematostella_vectensis/21-315 GS............QETETD..........FLSEVVLEF...........SEYISKNSKQ
Mus_musculus/396-579
150 160
Mus_musculus/396-579 ........RAQAAEAASPAG.......................GLED.L............
Homo_sapiens/395-577 ........RALAPGRASLGS.......................RLEDVL............
Chelonia_mydas/398-611 ........TAVSEDGARSSE....................NLWQGDHL............
Rhinatrema_bivittatum/70-211 ........YDTFKDGSLQGQ.........................QEDKL...........
Danio_rerio/391-562 ........TEDKPSVKLINE.........................QVEEQ...........
Latimeria_chalumnae/391-634 ........CAAAGGGSRSDHGS.................VHQEQEDDW...........
Aplysia_californica/466-956 .DDITDVMRSCSVEESTSSEICIDAKRFKPRGEKESPDLVNCDVLDELQFSQDSLNFSED
Patiria_miniata/30-206 ........SVKSFEVSQSKS.........................MLEDE...........
Nematostella_vectensis/21-315 ADP.....TGLEFHTEKTDE....................NLLKEDDLE.TKDALH....
α8
Mus_musculus/396-579
170 180
Mus_musculus/396-579 ....WLQEVSNLSEWLNPGHRS
Homo_sapiens/395-577 ....WLQEVSNLSEWLSPSPGP
Chelonia_mydas/398-611 ....WLQEISNLSEWTS.....
Rhinatrema_bivittatum/70-211 ....WLQEVSKLSDWTC.....
Danio_rerio/391-562 ....WLQELENFSEW......D
Latimeria_chalumnae/391-634 ....WLQEVLRLSEWTT.....
Aplysia_californica/466-956 GEEDVLKHWQRLSPLSGQLELNAAEHRSKTSQLDDSEDWLNE.EEFSKWQAN..MT
Patiria_miniata/30-206 ....WLDEVLDSDPFFNE...S
Nematostella_vectensis/21-315 ..KDVLN..............EERGIEKE..NSFDWLEEFENN F AVN....

Supplementary Figure 3. Conservation of TOPOVIBL C-terminal region

Alignment of TOPOVIBL C-terminal region from nine metazoan species (as in Fig. 1f). The region aligned starts from the three conserved helices of the transducer domain (α5, α6 and α7) up to the C-terminal end. The position of S452 in *M. musculus* TOPOVIBL which defines the N-terminal boarder of the protein used in biochemical assays (452-579) is shown by an arrow. Brown letters: equivalent amino acids; white letters: identical amino acids.

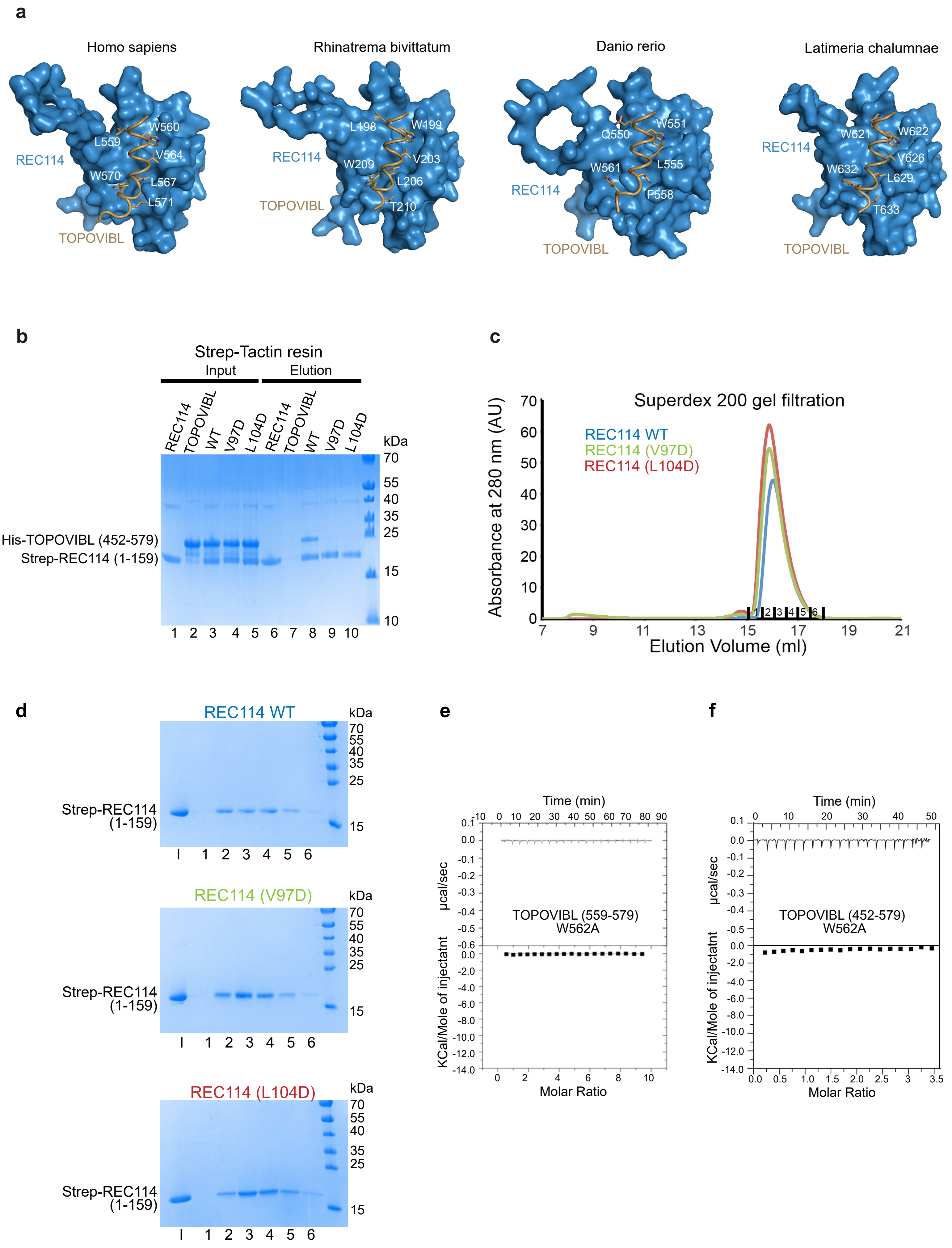
a
Homo sapiens
Rhinatrema bivittatum
Danio rerio
Latimeria chalumnae
REC114
TOPOVIBL
L559
W560
V564
W570
L567
L571
L198
W199
W209
V203
L206
T210
Q550
W551
W561
L555
F558
W621
W622
W632
V626
L629
T633
b
Strep-Tactin resin
Input
Elution
REC114
TOPOVIBL
WT
V97D
L104D
kDa
70
55
40
35
25
15
10
His-TOPOVIBL (452-579)
Strep-REC114 (1-159)
1 2 3 4 5 6 7 8 9 10
c
Superdex 200 gel filtration
REC114 WT
REC114 (V97D)
REC114 (L104D)
Absorbance at 280 nm (AU)
Elution Volume (ml)
d
REC114 WT
REC114 (V97D)
REC114 (L104D)
Strep-REC114 (1-159)
I 1 2 3 4 5 6
e
Time (min)
µcal/sec
TOPOVIBL (559-579) W562A
KCal/Mole of injectatnt
Molar Ratio
f
Time (min)
µcal/sec
TOPOVIBL (452-579) W562A
KCal/Mole of injectatnt
Molar Ratio

Supplementary Figure 4. Properties of the TOPOVIBL-REC114 complex and mutants

a. The TOPOVIBL-REC114 complex models predicted by AlphaFold2[29]. The structure of the REC114 PH domain is shown as surface in blue. Key interacting residues of TOPOVIBL are shown as sticks. Most of the interactions observed in the mouse crystal structure are conserved in the predicted complexes of other species. In *D. rerio*, the residue corresponding to mouse Val566 is Leu555. This mutation seems to be compensated by the valine to glutamine mutation at position 92 in REC114.

b. Pull-down experiments of Strep-tagged REC114$^{1\text{-}159}$ mutants indicated above the lanes with TOPOVIBL$^{452\text{-}579}$. All proteins were first purified by affinity chromatography and gel filtration. A total of 1.2% of the input (lanes 1–5) and 1.2% of the eluates (lanes 6–10) were analyzed on 12% SDS-PAGE gels stained with coomassie brilliant blue. Control lanes 1-2 and 6-7 show inputs and elutions of REC114 and TOPOVIBL alone, respectively.

c. Superdex 200 gel filtration elution profiles of WT and mutant Strep-REC114$^{1\text{-}159}$. The elution profiles are similar, indicating that the REC114 mutations do not significantly affect the overall structure of this REC114 fragment.

d. SDS-PAGE analysis of fractions 1-6 of the Superdex 200 gel filtration elution profiles shown in **c**. I indicates input samples loaded onto the column.

e. ITC measurement of the interaction affinity between REC114$^{15\text{-}159}$ and the TOPOVIBL$^{559\text{-}576}$ peptide harbouring the W562A mutation (the wild-type control is in Fig. 1g).

f. ITC measurement of the interaction affinity between REC114$^{1\text{-}159}$ and TOPOVIBL$^{452\text{-}579}$ (W562A) (the wild-type control is in Fig. 1g). Source data are provided as a Source Data file.

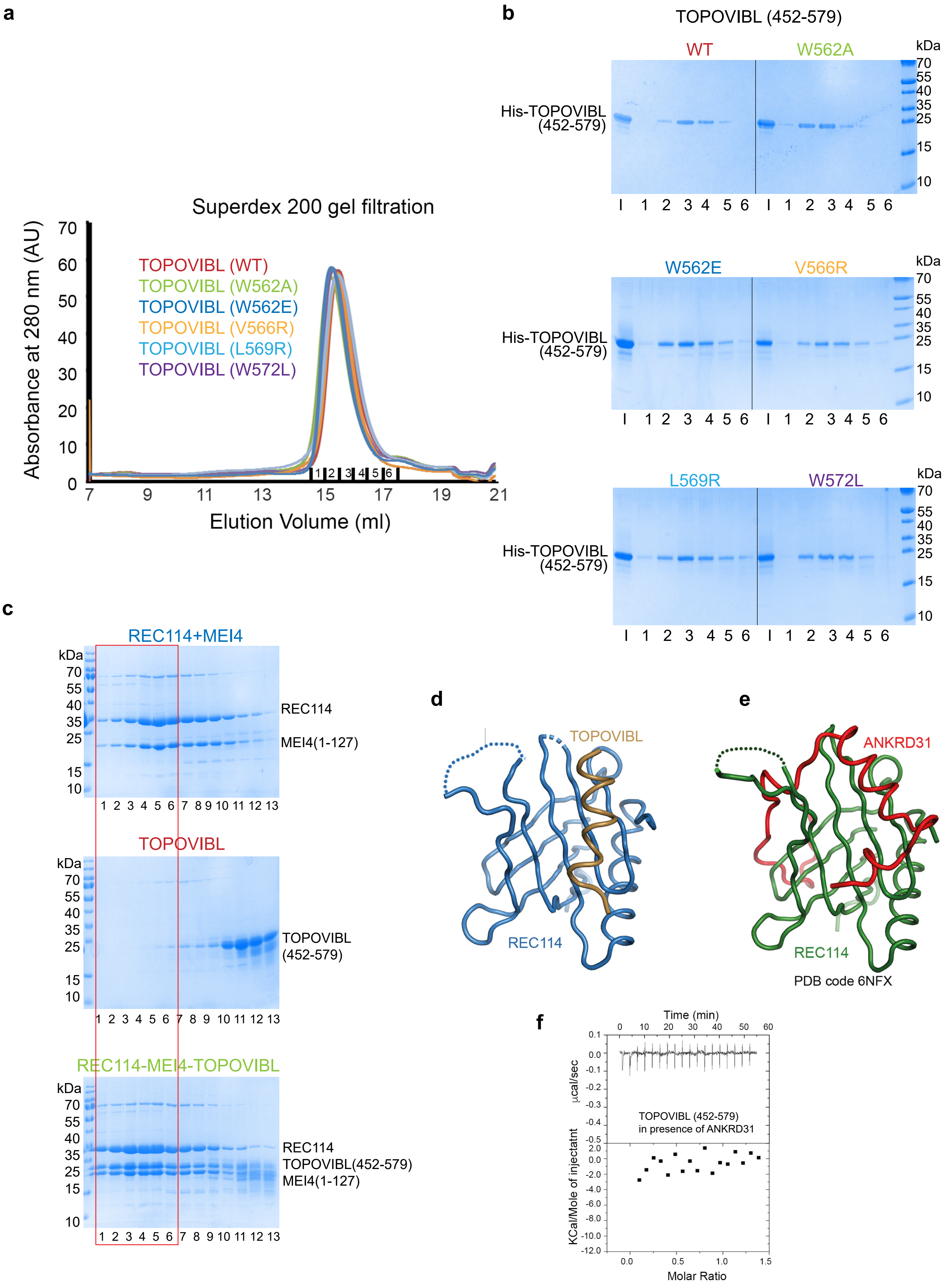

a
Superdex 200 gel filtration
Absorbance at 280 nm (AU)
70
60
50
40
30
20
10
0
7 9 11 13 15 17 19 21
Elution Volume (ml)
TOPOVIBL (WT)
TOPOVIBL (W562A)
TOPOVIBL (W562E)
TOPOVIBL (V566R)
TOPOVIBL (L569R)
TOPOVIBL (W572L)
1 2 3 4 5 6
b
TOPOVIBL (452-579)
WT
W562A
kDa
70 55 40 35 25 15 10
His-TOPOVIBL (452-579)
I 1 2 3 4 5 6 I 1 2 3 4 5 6
W562E
V566R
kDa
70 55 40 35 25 15 10
His-TOPOVIBL (452-579)
I 1 2 3 4 5 6 I 1 2 3 4 5 6
L569R
W572L
kDa
70 55 40 35 25 15 10
His-TOPOVIBL (452-579)
I 1 2 3 4 5 6 I 1 2 3 4 5 6
c
REC114+MEI4
kDa
70 55 40 35 25 15 10
REC114
MEI4(1-127)
1 2 3 4 5 6 7 8 9 10 11 12 13
TOPOVIBL
kDa
70 55 40 35 25 15 10
TOPOVIBL (452-579)
1 2 3 4 5 6 7 8 9 10 11 12 13
REC114-MEI4-TOPOVIBL
kDa
70 55 40 35 25 15 10
REC114
TOPOVIBL(452-579)
MEI4(1-127)
1 2 3 4 5 6 7 8 9 10 11 12 13
d
TOPOVIBL
REC114
e
ANKRD31
REC114
PDB code 6NFX
f
Time (min)
0 10 20 30 40 50 60
µcal/sec
0.1 0.0 -0.1 -0.2 -0.3 -0.4 -0.5
TOPOVIBL (452-579)
in presence of ANKRD31
KCal/Mole of injectatnt
2.0 0.0 -2.0 -4.0 -6.0 -8.0 -10.0 -12.0
0.0 0.5 1.0 1.5
Molar Ratio

Supplementary Figure 5. Properties of the TOPOVIBL-REC114-MEI4 complex and mutants

a. Superdex 200 gel filtration elution profiles of WT and mutant TOPOVIBL$^{452-579}$. The elution profiles are similar, indicating that the TOPOVIBL mutations do not significantly affect the overall structure of this TOPOVIBL fragment.

b. SDS-PAGE analysis of fractions 1-6 of the Superdex 200 gel filtration elution profiles shown in **a**. I indicates input samples loaded onto the column.

c. SDS-PAGE analysis of fractions 1-13 of the Superose 6 gel filtration elution profiles shown in Fig. 2i. The red rectangle highlights fractions of containing the TOPOVIBL-REC114-MEI4 complex.

d. and e. Comparison of the REC114-TOPOVIBL (d) and the REC114-ANKRD31 (e) structures.

f. ITC measurement of the interaction between REC114$^{15-159}$ and TOPOVIBL$^{452-579}$ shows the absence of binding when REC114 is pre-saturated with ANKRD31$^{1808-1857}$. Source data are provided as a Source Data file.

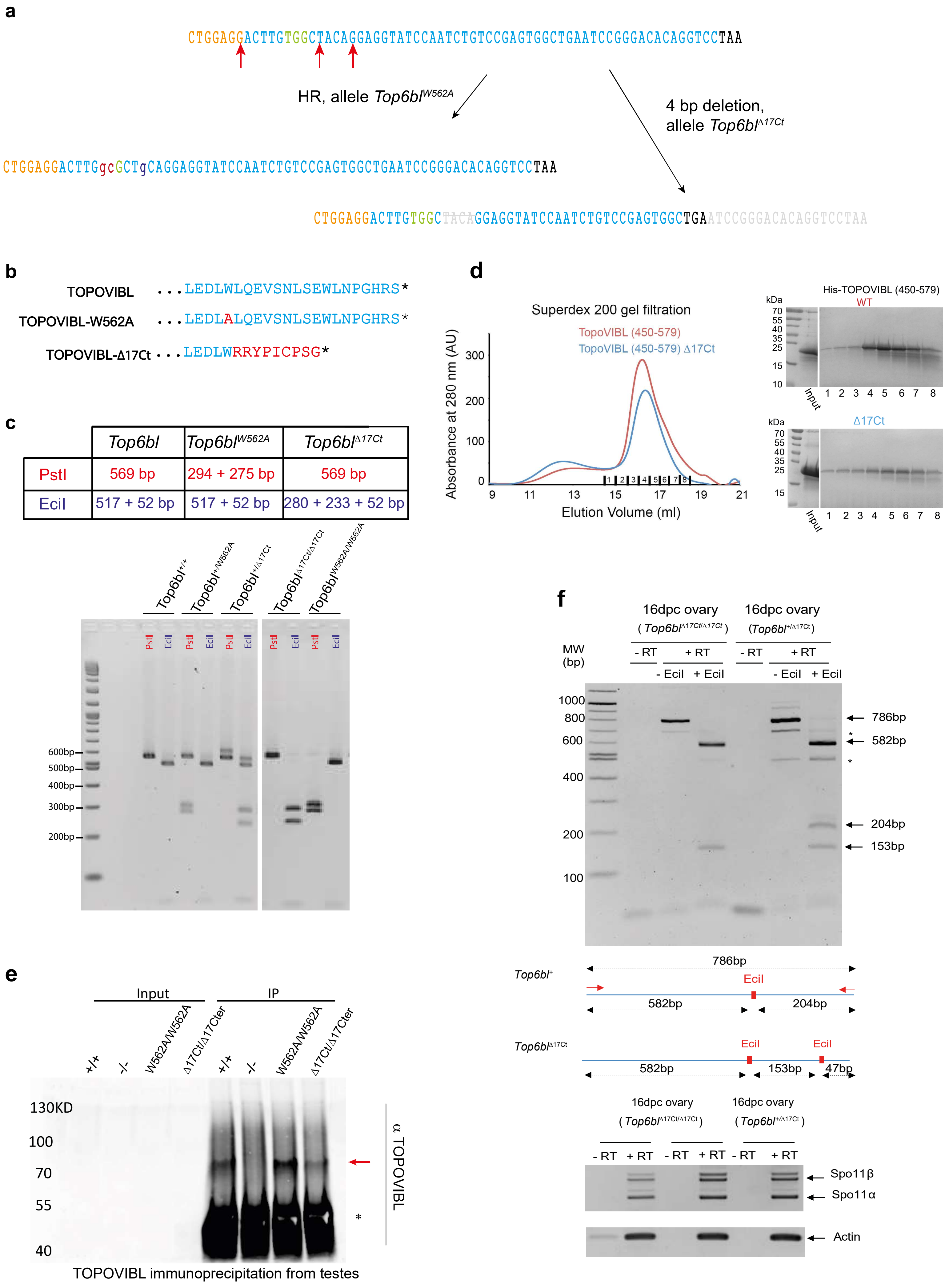
a
CTGGAGGACTTGTGGCTACAGGAGGTATCCAATCTGTCCGAGTGGCTGAATCCGGGACACAGGTCCTAA
HR, allele Top6bl W562A
4 bp deletion, allele Top6bl Δ17Ct
CTGGAGGACTTGgcGCTgCAGGAGGTATCCAATCTGTCCGAGTGGCTGAATCCGGGACACAGGTCCTAA
CTGGAGGACTTGTGGCTACAGGAGGTATCCAATCTGTCCGAGTGGCTGAATCCGGGACACAGGTCCTAA
b
TOPOVIBL ...LEDLWLQEVSNLSEWLNPGHRS*
TOPOVIBL-W562A ...LEDLALQEVSNLSEWLNPGHRS*
TOPOVIBL-Δ17Ct ...LEDLWRRYPICPSG*
c
Top6bl | Top6bl W562A | Top6bl Δ17Ct
PstI | 569 bp | 294 + 275 bp | 569 bp
EciI | 517 + 52 bp | 517 + 52 bp | 280 + 233 + 52 bp
Top6bl +/+
Top6bl +/W562A
Top6bl +/Δ17Ct
Top6bl Δ17Ct/Δ17Ct
Top6bl W562A/W562A
PstI
EciI
600bp
500bp
400bp
300bp
200bp
d
Superdex 200 gel filtration
TopoVIBL (450-579)
TopoVIBL (450-579) Δ17Ct
Absorbance at 280 nm (AU)
Elution Volume (ml)
His-TOPOVIBL (450-579)
WT
Δ17Ct
kDa
Input
e
Input
IP
+/+
-/-
W562A/W562A
Δ17Ct/Δ17Cter
130KD
100
70
55
40
α TOPOVIBL
TOPOVIBL immunoprecipitation from testes
f
16dpc ovary (Top6bl Δ17Ct/Δ17Ct)
16dpc ovary (Top6bl +/Δ17Ct)
MW (bp)
- RT
+ RT
- EciI
+ EciI
1000
800
600
400
200
100
786bp
582bp
204bp
153bp
Top6bl +
Top6bl Δ17Ct
EciI
47bp
Spo11β
Spo11α
Actin

Supplementary Figure 6. Generation and characterization of mice carrying *Top6bl*^{W562A} and *Top6bl*$^{\Delta 17Ct}$ alleles.

a. *Top6bl*, *Top6bl*^{W562A} and *Top6bl*$^{\Delta 17Ct}$ cDNA sequences. The top sequence corresponds to the 65 last nucleotides of the *Top6bl* open reading frame. Orange and blue letters mark exons 15 and 16, respectively. The TGG codon in green corresponds to the W562 residue in the protein sequence. Red arrows mark the position of the predicted CRISPR-Cas9 cleavage sites, corresponding to the three guide RNAs used. TAA in black: original stop codon.

The middle sequence corresponds to the repair product by homologous recombination (HR) following CRISPR-Cas9-mediated DSB formation. The TGG codon is replaced by the GCG codon (red letters). The G residue in purple corresponds to a silent substitution to create a *Pst*I restriction site for genotyping. TAA in black: original stop codon.

The bottom sequence corresponds to the product of the deletion of four nucleotides in the sequence following DSB formation and repair (grey crossed letters). TGA in black: new stop codon due to frameshift.

b. Protein sequences of the C-terminal part of TOPOVIBL, TOPOVIBL-W562A and TOPOVIBL-Δ17Ct. Blue letters correspond to the original residues, bold red A to the mutant substitution of W562 in the TOPOVIBL-W562A sequence, and red letters to the nine-residue substitution in the TOPOVIBL-Δ17Ct sequence. *: Stop.

c. Genotyping strategy. Following DNA extraction from tails of *Top6bl*$^{+/+}$, *Top6bl*$^{+/W562A}$, *Top6bl*$^{+/\Delta 17Ct}$, *Top6bl*$^{W562A/W562A}$ and *Top6bl*$^{\Delta 17Ct/\Delta 17Ct}$ mice, PCR amplification with the primer pair specific for a 569 bp region containing the mutations was performed. PCR products were digested with *Pst*I or *Eci*I. The top table presents the expected size following digestion of the different alleles (red correspond to *Pst*I digestion, and dark blue to *Eci*I digestion). Bottom panel: DNA fragments separated on agarose gels.

d. Left panel: Overlay of the Superdex 200 gel filtration elution profiles of wild-type TOPOVIBL$^{452-579}$ and TOPOVIBL$^{52-579}$ Δ17Ct. Both profiles are similar, indicating that the Δ17Ct mutation does not significantly affect the overall structure of this TOPOVIBL fragment. Right panel: SDS-PAGE analysis of the input and fractions 1-8 of the Superdex 200 gel filtration elution profiles. Gels were stained with coomassie brilliant blue.

e. TOPOVIBL immunoprecipitation. Following extraction of total protein from 14 dpp mouse testes obtained from *Top6bl*$^{+/+}$, *Top6bl*$^{W562A/W562A}$ *Top6bl*$^{\Delta17Ct/\Delta17Ct}$ and *Top6bl*$^{-/-}$ mice, TOPOVIBL was immunoprecipitated using the homemade anti-TOPOVIBL antibody. Six testes of each genotype were used for one immunoprecipitation. The immunoblot was revealed using the homemade anti-TOPOVIBL antibody. The red arrow shows the TOPOVIBL expected band. *corresponds to a non-specific band (immunoglobulin heavy chain).

f. Expression of *Top6bl* assessed by RT-PCR. RNA extracted from ovaries of 16 dpc *Top6bl* $^{\Delta17Ct/\Delta17Ct}$ or *Top6bl* $^{+/\Delta17Ct}$ mice was incubated with or without reverse transcriptase (RT), amplified by PCR and incubated with or without *Eci*I restriction enzyme. The size of the fragments after *Eci*I digestion are shown for the *Top6bl* $^{+}$ and *Top6bl* $^{\Delta17Ct}$ alleles. Stars indicate non-specific amplification products. Control PCR assays for monitoring the expression of the two *Spo11* splice variants (*Spo11*α and β) and of actin are shown. Source data are provided as a Source Data file.

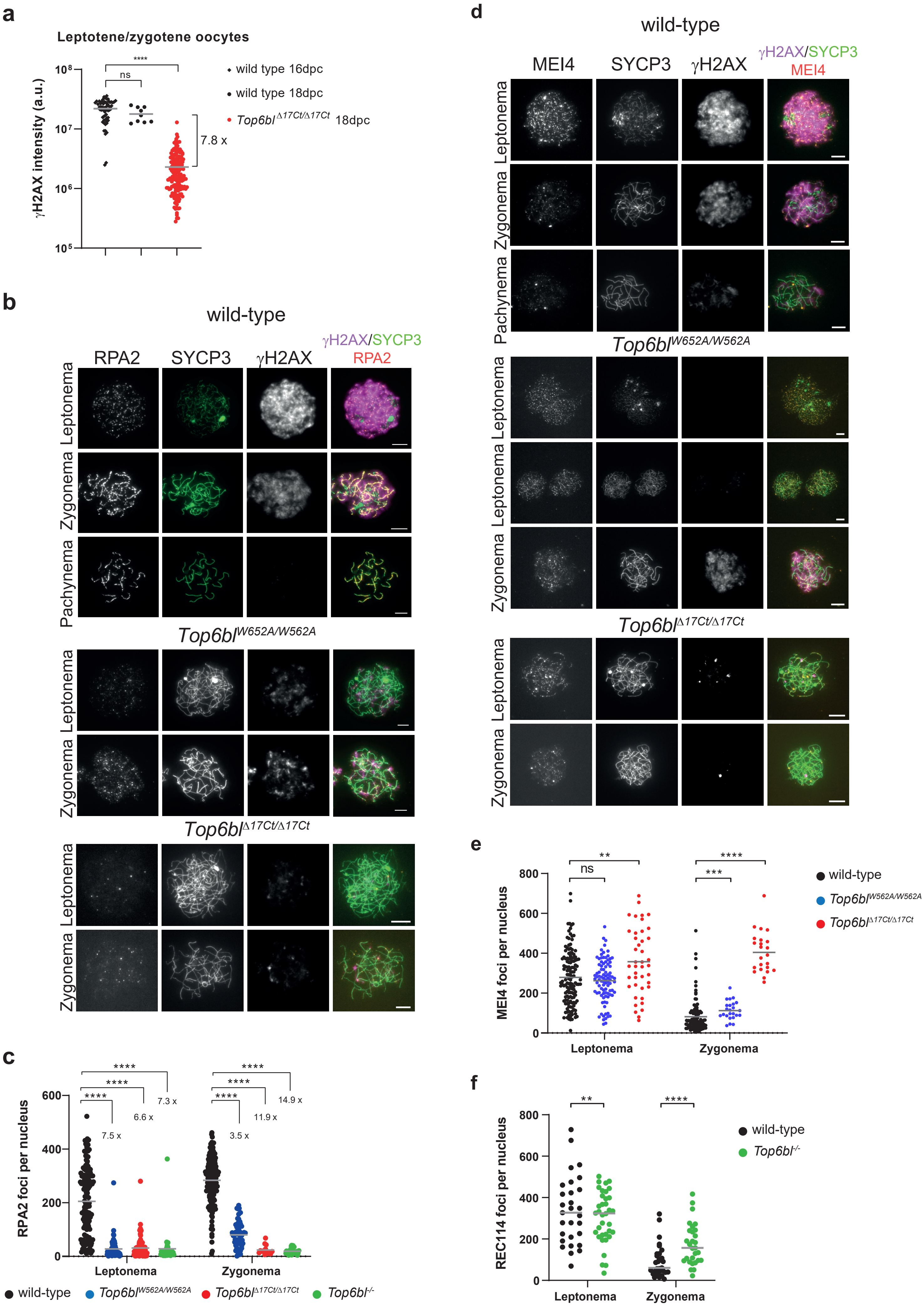
a
Leptotene/zygotene oocytes
γH2AX intensity (a.u.)
****
ns
7.8 x
wild type 16dpc
wild type 18dpc
Top6bl^Δ17Ct/Δ17Ct 18dpc
b
wild-type
RPA2
SYCP3
γH2AX
γH2AX/SYCP3
RPA2
Leptonema
Zygonema
Pachynema
Top6bl^W652A/W562A
Leptonema
Zygonema
Top6bl^Δ17Ct/Δ17Ct
Leptonema
Zygonema
c
RPA2 foci per nucleus
600
400
200
0
****
7.5 x
6.6 x
7.3 x
3.5 x
11.9 x
14.9 x
Leptonema
Zygonema
wild-type
Top6bl^W562A/W562A
Top6bl^Δ17Ct/Δ17Ct
Top6bl^-/-
d
wild-type
MEI4
SYCP3
γH2AX
γH2AX/SYCP3
MEI4
Leptonema
Zygonema
Pachynema
Top6bl^W652A/W562A
Leptonema
Leptonema
Zygonema
Top6bl^Δ17Ct/Δ17Ct
Leptonema
Zygonema
e
MEI4 foci per nucleus
800
600
400
200
0
**
ns
****
***
Leptonema
Zygonema
wild-type
Top6bl^W562A/W562A
Top6bl^Δ17Ct/Δ17Ct
f
REC114 foci per nucleus
800
600
400
200
0
**
****
Leptonema
Zygonema
wild-type
Top6bl^-/-

Supplementary Figure 7. Immunocytochemistry on oocytes

a. Quantification of γH2AX signal intensity in leptotene (L)/zygotene (Z) oocytes from 16 and 18 dpc wild-type and 18dpc *Top6bl* $^{\Delta 17Ct/\Delta 17Ct}$ mice. Number of nuclei: 64, 9, and 167, respectively. Grey bars show the mean values. P values were determined using the two-tailed unpaired Mann-Whitney test. Progression into prophase at 18dpc was: L 46%, Z 54%, Pachynema (P) 0% in *Top6bl* $^{\Delta 17Ct/\Delta 17Ct}$, and L 2%, Z 8%, P 90% in wild-type oocytes.

b. Immunostaining of RPA2, SYCP3 and γH2AX in oocytes from 16 dpc wild-type (+/+), *Top6bl*$^{W562A/W562A}$, and *Top6bl* $^{\Delta 17Ct/\Delta 17Ct}$ ovaries. Scale bar, 10μm.

c. Quantification of RPA foci per nucleus in leptotene and zygotene oocytes from 16 dpc wild-type (+/+ or *Top6bl* $^{+/\Delta 17Ct}$), *Top6bl*$^{W562A/W562A}$, *Top6bl* $^{\Delta 17Ct/\Delta 17Ct}$, and *Top6bl*$^{-/-}$ mice (n=4 wild-type and n=2 mice per mutant genotype). Number of nuclei per genotype at leptonema (157, 85, 14, and 29) and at zygonema (165, 55, 105, and 28). Grey bars show the mean value. P values were determined using the two-tailed unpaired Mann-Whitney test. Ratios of the wild-type and mutant mean values are shown.

d. Immunostaining of MEI4, SYCP3 and γH2AX in oocytes from 16 dpc wild-type (+/+), *Top6bl*$^{W562A/W562A}$, and *Top6bl* $^{\Delta 17Ct/\Delta 17Ct}$ ovaries. Scale bar, 10μm.

e. Quantification of axis-associated MEI4 foci in leptotene and zygotene oocytes from 15 and 16 dpc wild-type (+/+ or *Top6bl* $^{+/\Delta 17Ct}$), *Top6bl*$^{W562A/W562A}$, and *Top6bl* $^{\Delta 17Ct/\Delta 17Ct}$ mice (n=3 wild-type, n=1 *Top6bl*$^{W562A/W562A}$, and n=2 *Top6bl* $^{\Delta 17Ct/\Delta 17Ct}$ mice). Number of nuclei per genotype at leptonema (139, 91, and 42) and at zygonema (106, 24, and 23). Grey bars show the mean values. P values were determined using the two-tailed unpaired Mann-Whitney test.

f. Quantification of axis-associated REC114 foci in leptotene and zygotene oocytes from 16 dpc wild-type (+/+) and *Top6bl*$^{-/-}$ mice (n=1 mouse/genotype). Number of nuclei: 29 and 36 L, and 31 and 29 Z, respectively. Grey bars show the mean values. P values were determined using the two-tailed unpaired Mann-Whitney test. Source data are provided as a Source Data file.

a

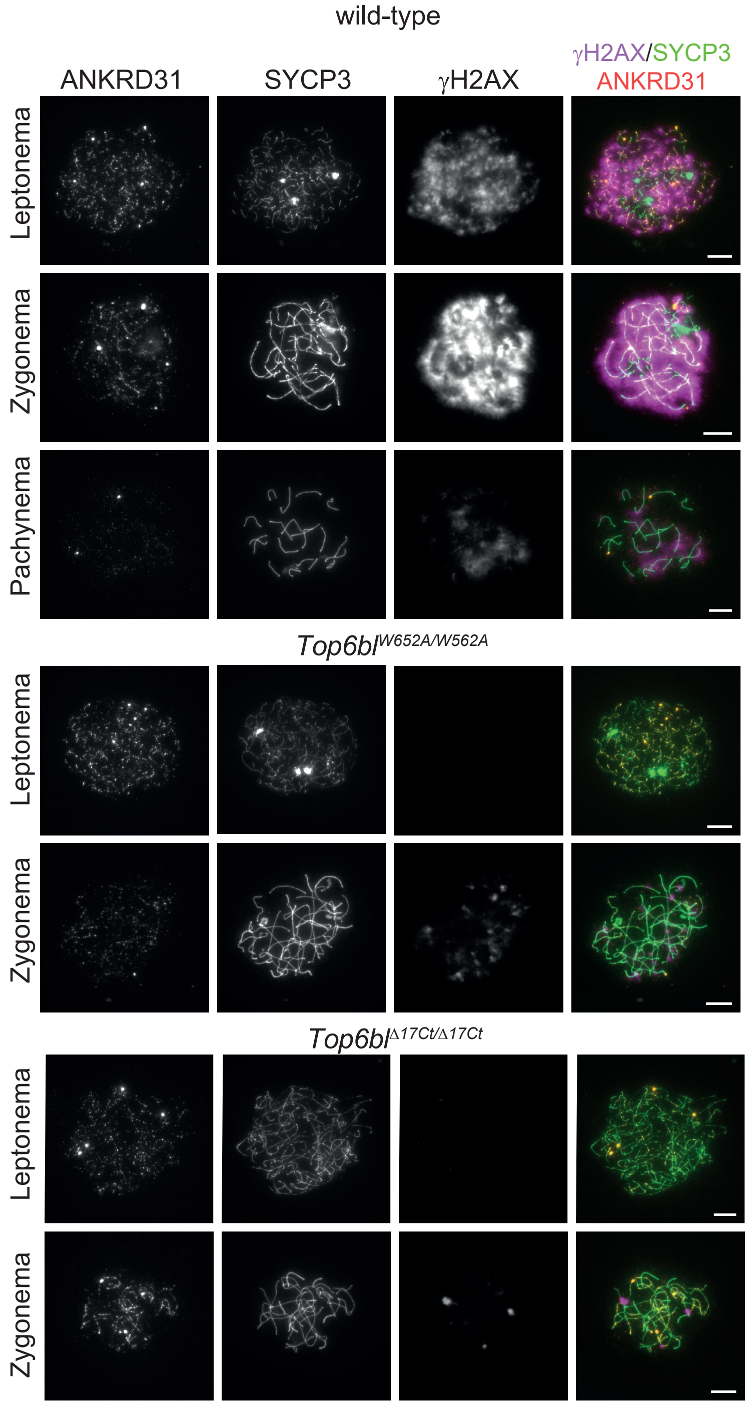

wild-type
ANKRD31
SYCP3
γH2AX
γH2AX/SYCP3
ANKRD31
Leptonema
Zygonema
Pachynema
Top6bl W652A/W562A
Leptonema
Zygonema
Top6bl Δ17Ct/Δ17Ct
Leptonema
Zygonema

b

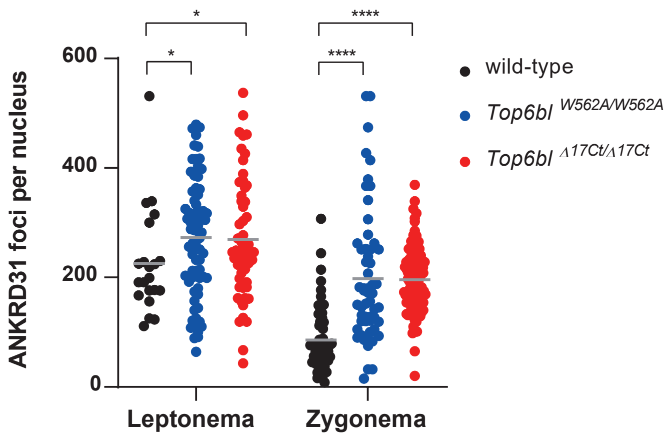

ANKRD31 foci per nucleus
600
400
200
0
*
*
****
****
Leptonema
Zygonema
wild-type
Top6bl W562A/W562A
Top6bl Δ17Ct/Δ17Ct

Supplementary Figure 8. Immunocytochemistry of ANKRD31 on oocytes

a. Immunostaining of ANKRD31, SYCP3 and γH2AX in oocytes from 16 dpc wild-type (+/+), *Top6bl*$^{W562A/W562A}$, and *Top6bl* $^{Δ17Ct/Δ17Ct}$ ovaries. Scale bar, 10μm.

b. Quantification of axis-associated ANKRD31 foci in leptotene and zygotene oocytes from 16 and 18 dpc wild-type (+/+ or *Top6bl* $^{+/Δ17Ct}$), *Top6bl*$^{W562A/W562A}$, and *Top6bl* $^{Δ17Ct/Δ17Ct}$ mice (n=3 wild-type, n=1 *Top6bl*$^{W562A/W562A}$, and n=2 *Top6bl* $^{Δ17Ct/Δ17Ct}$ mice). Number of nuclei per genotype at leptonema (21, 74, and 56) and at zygonema (56, 57, and 108). Grey bars show the mean values. P values were determined using the two-tailed unpaired Mann-Whitney test. Source data are provided as a Source Data file.

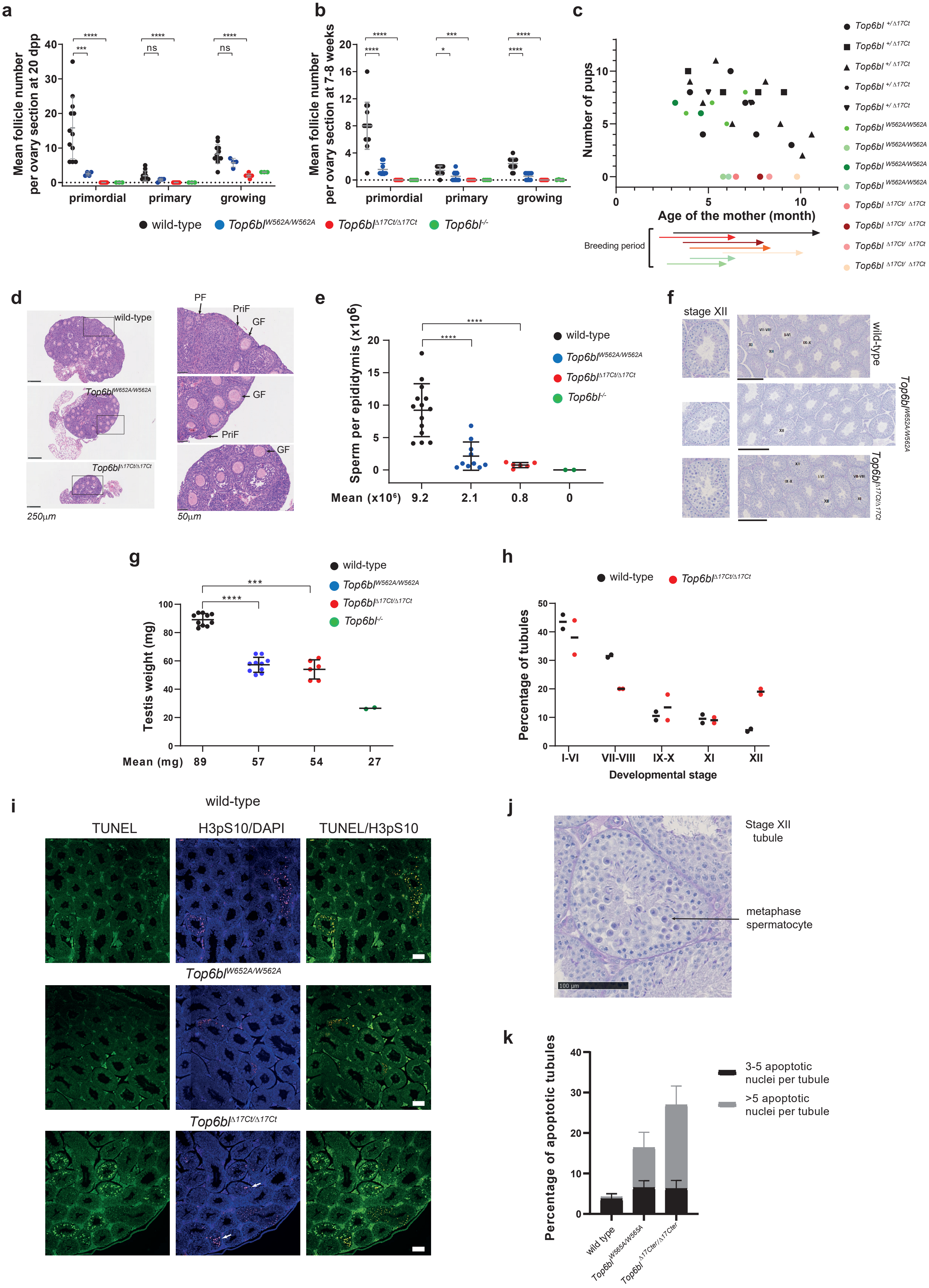
a
Mean follicle number per ovary section at 20 dpp
primordial
primary
growing
ns
wild-type
Top6bl W562A/W562A
Top6bl Δ17Ct/Δ17Ct
Top6bl -/-
b
Mean follicle number per ovary section at 7-8 weeks
c
Number of pups
Age of the mother (month)
Breeding period
Top6bl +/Δ17Ct
Top6bl W562A/W562A
Top6bl Δ17Ct/ Δ17Ct
d
wild-type
Top6bl W652A/W562A
Top6bl Δ17Ct/Δ17Ct
PF
PriF
GF
250µm
50µm
e
Sperm per epididymis (x10⁶)
Mean (x10⁶) 9.2 2.1 0.8 0
f
stage XII
wild-type
Top6bl W652A/W562A
Top6bl Δ17Ct/Δ17Ct
g
Testis weight (mg)
Mean (mg) 89 57 54 27
h
Percentage of tubules
Developmental stage
I-VI
VII-VIII
IX-X
XI
XII
i
wild-type
TUNEL
H3pS10/DAPI
TUNEL/H3pS10
Top6bl W652A/W562A
Top6bl Δ17Ct/Δ17Ct
j
Stage XII tubule
metaphase spermatocyte
100 µm
k
Percentage of apoptotic tubules
3-5 apoptotic nuclei per tubule
>5 apoptotic nuclei per tubule
wild type
Top6bl W565A/W565A
Top6bl Δ17Cter/Δ17Cter

Supplementary Figure 9. Histology on ovaries and testis

a. Quantification of primordial, primary, and growing follicles in ovaries from 20 dpp wild-type homozygous or heterozygous (+/+, *Top6bl* $^{+/\Delta 17Ct}$, *Top6bl* $^{+/W562A}$, +/ *Top6bl*$^{-}$), *Top6bl*$^{W562A/W562A}$, *Top6bl* $^{\Delta 17Ct/\Delta 17Ct}$, and *Top6bl*$^{-/-}$ mice. Each data point is the mean ± SD of at least 10 sections from one ovary. Number of mice: 6, 2, 4, and 2, respectively. P values were determined using the two-tailed unpaired Mann-Whitney test.

b. Quantification of primordial, primary, and growing follicles in ovaries from 7-8-week-old wild-type (+/+ or *Top6bl* $^{+/W562A}$), *Top6bl*$^{W562A/W562A}$, *Top6bl* $^{\Delta 17Ct/\Delta 17Ct}$, and *Top6bl*$^{-/-}$ mice. Each data point is the mean ± SD of 20 sections from one ovary. P values were determined using the two-tailed unpaired Mann-Whitney tests. Number of mice: 7, 5, 3, and 2 respectively.

c. Fertility tests. *Top6bl* $^{+/\Delta 17Ct}$, *Top6bl*$^{W562A/W562A}$ and *Top6bl* $^{\Delta 17Ct/\Delta 17Ct}$ females (n= 5, 4 and 4 respectively), from 2.3 to 10.6 months of age, were mated with heterozygous males (*Top6bl* $^{+/\Delta 17Ct}$ or *Top6bl* $^{+/W562A}$). Each symbol refers to a female. Only two of the four *Top6bl*$^{W562A/W562A}$ females were fertile (large circle, dark green, and small circle), and after the last progeny, they were not mated any longer. Breeding periods of non-productive females and for *Top6bl* $^{+/\Delta 17Ct}$ females are shown.

d. Haematoxylin and eosin-stained sections of ovaries from 20 dpp wild-type (+/+), *Top6bl*$^{W562A/W562A}$ and *Top6bl* $^{\Delta 17Ct/\Delta 17Ct}$ mice (samples from panel a). Scale bars, 250 and 50 μm in the left and right panel, respectively. Primordial (PriF), primary (PF), and growing (GF) follicles are indicated.

e. Sperm count in *Top6bl* mutants. Sperm in epididymis was counted in 2-month-old mice. Each data point is one epididymis (number of mice= 7, 5, 3 and 1 for wild-type (+/+ or +/-), *Top6bl*$^{W562A/W562A}$, *Top6bl* $^{\Delta 17Ct/\Delta 17Ct}$, and *Top6bl*$^{-/-}$, respectively). The mean ± SD is shown. P values were determined using the two-tailed unpaired Mann-Whitney test.

f. Periodic acid-Schiff staining of testis sections from 2 month-old wild-type (+/+), *Top6bl*$^{W562A/W562A}$, and *Top6bl* $^{\Delta 17Ct/\Delta 17Ct}$ mice. Scale bar, 250μm. Zoomed images of stage XII tubules are shown on the right.

g. Testis weight in *Top6bl* mutants. Testis weight was measured in 2 month-old mice. Each data point is one testis (number of mice= 5, 5, 3, and 1 for wild-type (+/+), *Top6bl*$^{W562A/W562A}$, *Top6bl* $^{\Delta 17Ct/\Delta 17Ct}$, and

Top6bl$^{-/-}$, respectively). The mean value ± SD is shown. P values were determined using the two-tailed unpaired Mann-Whitney test.

h. Staging of tubules in testis sections from 2-month-old wild-type and *Top6bl* $^{\Delta 17Ct/\Delta 17Ct}$ mice (n=2 mice/genotype). Tubules were divided in five groups based on several criteria. I-VI: round spermatid with acrosomes, 0 to ~25% of the perimeter of the spermatid, elongated spermatids on the lumen; VII-VIII: round spermatids with acrosomes, ~50% of the perimeter; IX-X: elongated spermatids with acrosomes not hook-shaped; XI: elongated spermatids with a hook; XII: presence of metaphases. Number of staged tubules: 310 in wild-type and 366 in *Top6bl* $^{\Delta 17Ct/\Delta 17Ct}$ mice. The horizontal bars show the mean value. The distributions are statistically different (Pearson's Chi-Square: 36.028).

i. Detection of apoptosis and metaphase in testis sections. Sections were labelled with TUNEL (green) and H3pS10 (red), and stained with DAPI (blue). Scale bar, 100µm. White arrowheads indicate tubules positive for H3pS10 and TUNEL.

j. A stage XII tubule with metaphase spermatocytes (from *Top6bl* $^{\Delta 17Ct/\Delta 17Ct}$ mice). Scale bar, 100µm. Several metaphases with condensed chromosomes are seen, and one (arrow) shows relatively more intense nuclear staining, suggesting apoptosis.

k. Detection of apoptosis by TUNEL assay. Tubules were classified in three categories: <3, 3 to 5, and >5 apoptotic nuclei. The mean values ± SD of the 3 to 5 and >5 categories are shown. Number of 2 month-old mice: 4 wild-type (+/+ and *Top6bl* $^{+/\Delta 17Ct}$) and 2 mice per mutant. The percentage of tubules with <3, 3 to 5, and >5 apoptotic nuclei was significatively different between wild-type and *Top6bl*$^{W562A/W562A}$ and *Top6bl* $^{\Delta 17Ct/\Delta 17Ct}$ mice (Chi-square Pearson: 354.03 and 811.63, respectively). Source data are provided as a Source Data file.

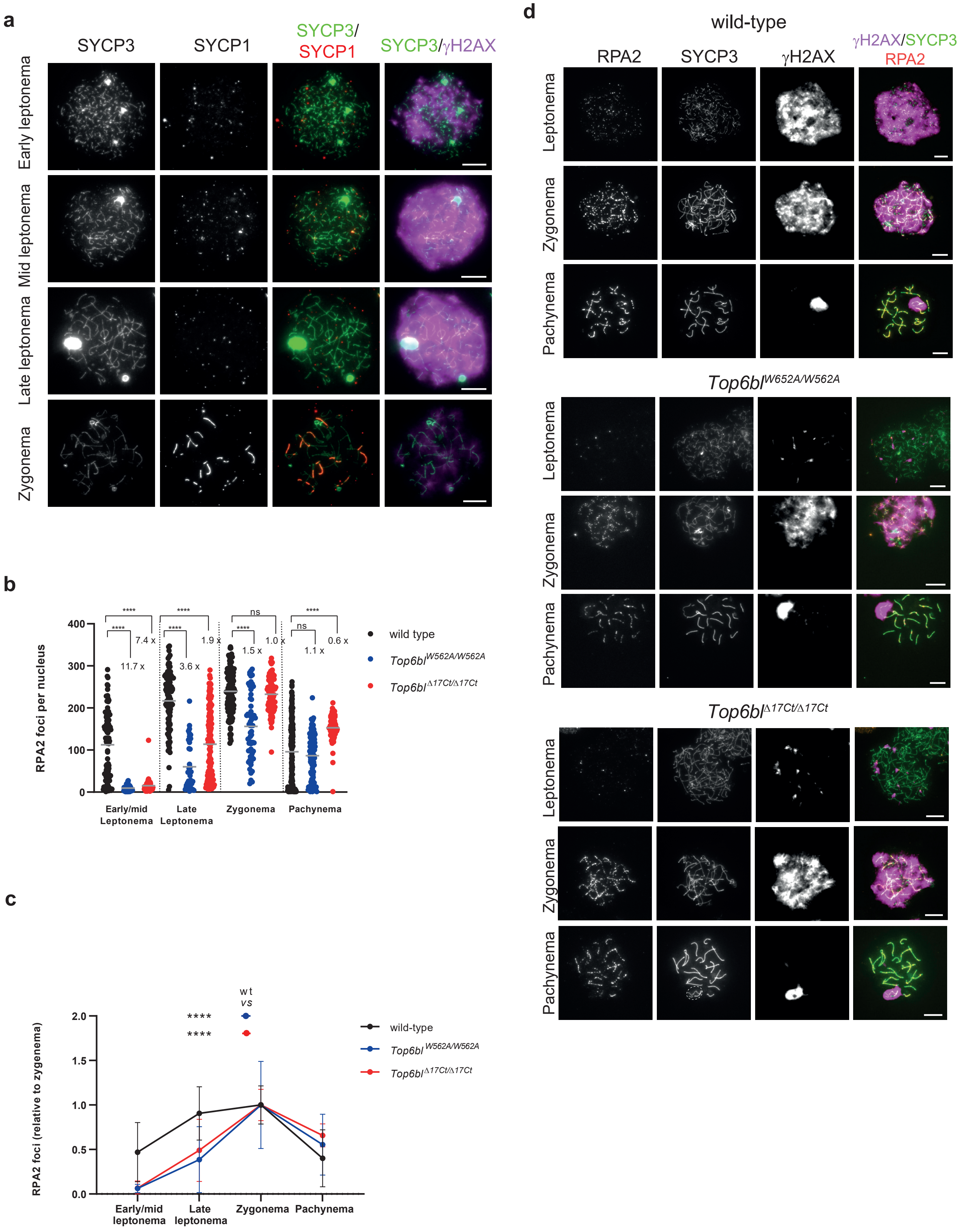
a
SYCP3
SYCP1
SYCP3/SYCP1
SYCP3/γH2AX
Early leptonema
Mid leptonema
Late leptonema
Zygonema
b
RPA2 foci per nucleus
400
300
200
100
0
****
****
7.4 x
11.7 x
3.6 x
1.9 x
1.5 x
1.0 x
ns
1.1 x
0.6 x
Early/mid Leptonema
Late Leptonema
Zygonema
Pachynema
wild type
Top6bl W562A/W562A
Top6bl Δ17Ct/Δ17Ct
c
RPA2 foci (relative to zygenema)
2.0
1.5
1.0
0.5
0.0
wt vs
****
****
Early/mid leptonema
Late leptonema
Zygonema
Pachynema
wild-type
Top6bl W562A/W562A
Top6bl Δ17Ct/Δ17Ct
d
wild-type
RPA2
SYCP3
γH2AX
γH2AX/SYCP3 RPA2
Leptonema
Zygonema
Pachynema
Top6bl W652A/W562A
Top6bl Δ17Ct/Δ17Ct

Supplementary Figure 10. Staging and immunocytochemistry on spermatocytes

a. Identification of spermatocytes at early, mid, late leptonema and zygonema. Examples of wild-type nuclei at the different stages by staining with SYCP3 (green), SYCP1 (red) and γH2AX (magenta). Scale bar, 10μm.

b. Quantification of axis-associated RPA2 foci in early/mid and late leptotene, zygotene and pachytene spermatocytes from 12-14dpp wild-type (+/+ or *Top6bl*$^{+/\Delta 17Ct}$; n=3), *Top6bl*$^{W562A/W562A}$ (n=2), and *Top6bl* $^{\Delta 17Ct/\Delta 17Ct}$ (n=2) mice. Number of nuclei per genotype at early/mid leptotene (105, 36, and 41), late leptotene (91, 38, and 131), zygotene (135, 62, and 85), and pachytene (178, 119, and 80). Grey bars show the mean values. P values were determined using the two-tailed unpaired Mann-Whitney test.

c. RPA2 focus variation during prophase. For each genotype (wild-type, *Top6bl*$^{W562A/W562A}$, and *Top6bl* $^{\Delta 17Ct/\Delta 17Ct}$), the number of RPA2 foci at the different stages (dataset as in panel b) was normalized to the mean number at zygonema (set to 1). The mean value ± SD is shown. Statistical significance between wild-type and each mutant (*Top6bl*$^{W562A/W562A}$; *Top6bl* $^{\Delta 17Ct/\Delta 17Ct}$) was tested at late leptonema using the two-tailed unpaired Mann-Whitney test.

d. Immunostaining of RPA2, SYCP3 and γH2AX in spermatocytes from 12-14dpp wild-type (+/+), *Top6bl*$^{W562A/W562A}$, and *Top6bl* $^{\Delta 17Ct/\Delta 17Ct}$ spermatocytes. A white dotted circle highlights the unsynapsed X and Y chromosomes at pachynema (SYCP3 staining) in *Top6bl* $^{\Delta 17Ct/\Delta 17Ct}$ mice. Scale bar, 10μm. Source data are provided as a Source Data file.

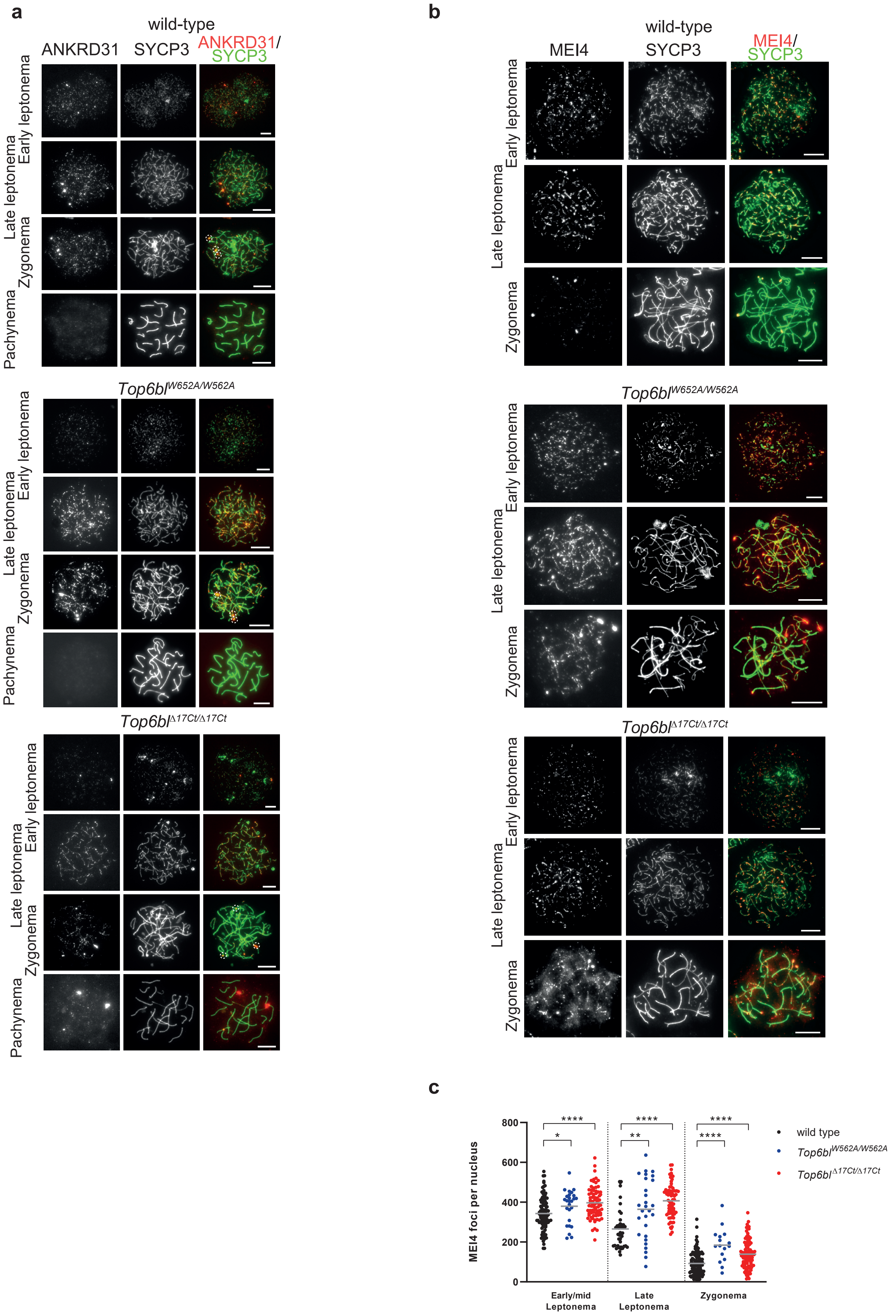
a
wild-type
ANKRD31
SYCP3
ANKRD31/SYCP3
Early leptonema
Late leptonema
Zygonema
Pachynema
Top6blW652A/W562A
Top6blΔ17Ct/Δ17Ct
b
wild-type
MEI4
SYCP3
MEI4/SYCP3
Early leptonema
Late leptonema
Zygonema
Top6blW652A/W562A
Top6blΔ17Ct/Δ17Ct
c
MEI4 foci per nucleus
800
600
400
200
0
****
*
**
****
****
****
Early/mid Leptonema
Late Leptonema
Zygonema
wild type
Top6blW562A/W562A
Top6blΔ17Ct/Δ17Ct

Supplementary Figure 11. Immunocytochemistry on spermatocytes

a. Immunostaining of ANKRD31 and SYCP3 in spermatocytes from 12-14dpp wild-type (+/+), *Top6bl*$^{W562A/W562A}$, and *Top6bl*$^{\Delta17Ct/\Delta17Ct}$ mice. At zygonema, dotted white circles (merge panels) highlight ANKRD31 aggregates at the ends of some chromosome axes. Scale bar, 10µm.

b. Immunostaining of MEI4 and SYCP3 in spermatocytes from 12-14 dpp wild-type (+/+), *Top6bl*$^{W562A/W562A}$, and *Top6bl*$^{\Delta17Ct/\Delta17Ct}$ mice. Scale bar, 10µm.

c. Quantification of axis-associated MEI4 foci in early/mid, late leptotene and zygotene spermatocytes from 12-14 dpp wild-type (+/+ or *Top6bl*$^{+/\Delta17Ct}$; n=2), *Top6bl*$^{W562A/W562A}$ (n=1), and *Top6bl*$^{\Delta17Ct/\Delta17Ct}$ (n=2) mice. Number of nuclei: 97 early/mid L, 43 late L, 114 Z in wild-type; 26 early/mid L, 28 late L and 16 Z in *Top6bl*$^{W562A/W562A}$; 77 early/mid L, 68 late L and 103 Z in *Top6bl*$^{\Delta17Ct/\Delta17Ct}$. Grey bars show the mean values. P values were determined using the two-tailed unpaired Mann-Whitney test. Source data are provided as a Source Data file.

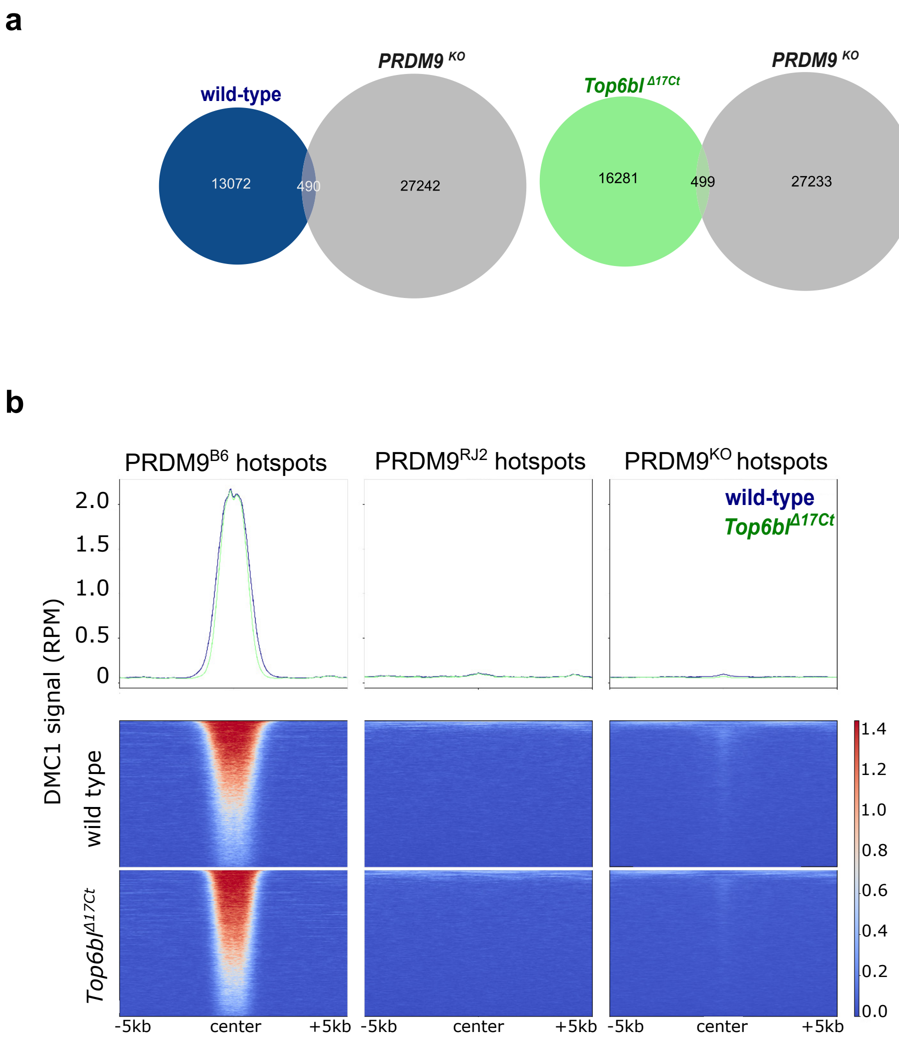

Supplementary Figure 12. Hotspot overlap analysis

a. Overlap between wild-type and *Top6bl*$^{\Delta17Ct/\Delta17Ct}$ hotspots and the default hotspots used in the *PRDM9*^{KO} mouse strain. The overlaps were restricted to the central 400bp around the estimated hotspot centre.

b. DMC1-SSDS signal (heatmap and average plots) from wild-type and *Top6bl*$^{\Delta17Ct/\Delta17Ct}$ mice at PRDM9^{B6}-, PRDM9^{RJ2}- and PRDM9^{KO}-defined hotspots.

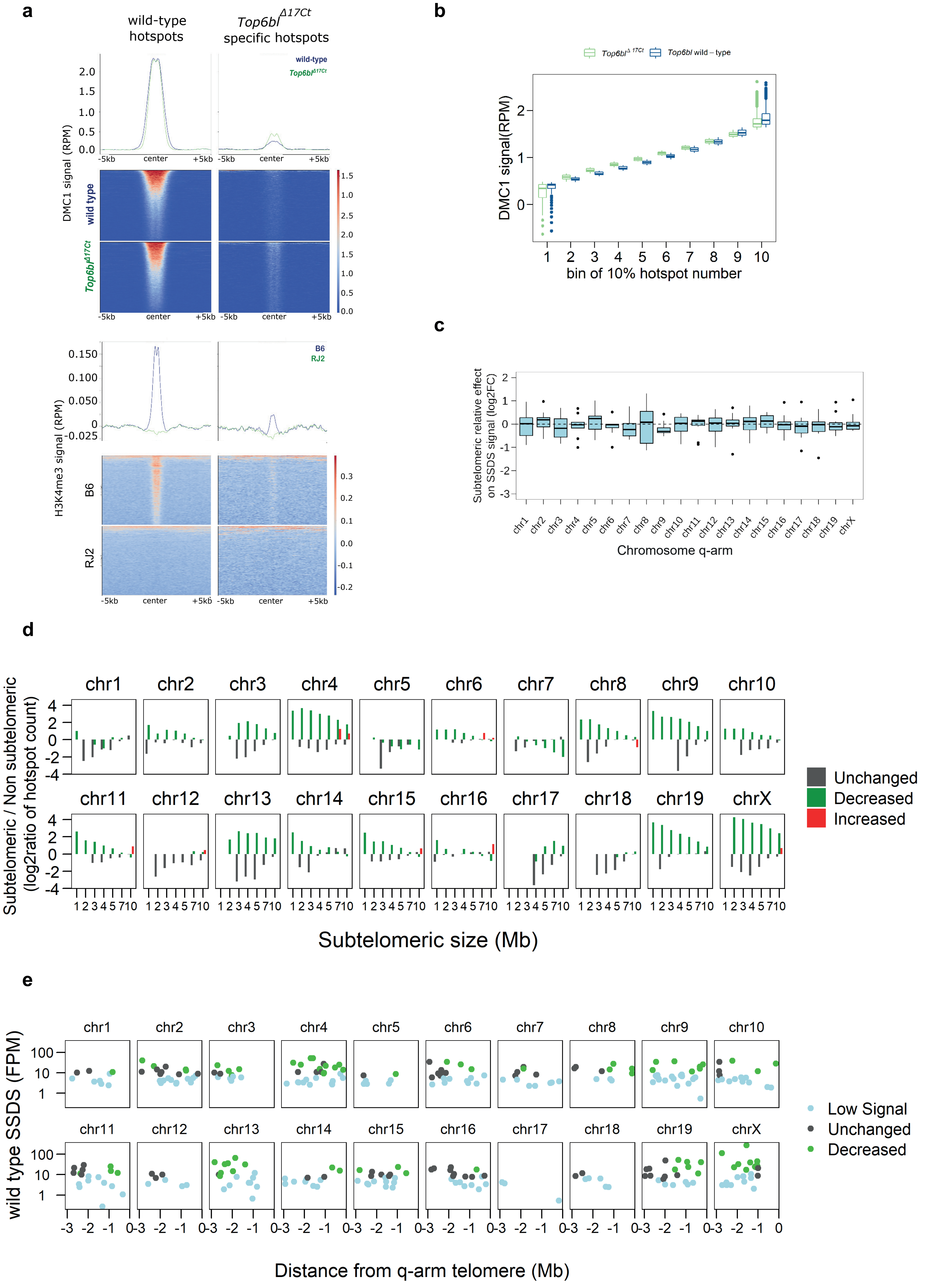
a
wild-type hotspots
Top6bl^Δ17Ct specific hotspots
wild-type
Top6bl^Δ17Ct
DMC1 signal (RPM)
wild type
Top6bl^Δ17Ct
-5kb center +5kb
H3K4me3 signal (RPM)
B6
RJ2
b
Top6bl^Δ17Ct Top6bl wild – type
DMC1 signal(RPM)
bin of 10% hotspot number
c
Subtelomeric relative effect on SSDS signal (log2FC)
Chromosome q-arm
d
Subtelomeric / Non subtelomeric (log2ratio of hotspot count)
Subtelomeric size (Mb)
Unchanged
Decreased
Increased
e
wild type SSDS (FPM)
Distance from q-arm telomere (Mb)
Low Signal
Unchanged
Decreased

Supplementary Figure 13. Hotspots signal quantification analysis

a. DMC1-SSDS signal from *Top6bl*$^{\Delta 17Ct/\Delta 17Ct}$ and wild-type mice at wild-type hotspots (13562) and at hotspots specifically activated in *Top6bl*$^{\Delta 17Ct/\Delta 17Ct}$ (3519), and H3K4me3 signal (heatmap and average plots) from B6 and RJ2 mice. Peaks are ranked as a function of the DMC1 or H3K4me3signal intensity.

b. DMC1-SSDS signal in *Top6bl*$^{\Delta 17Ct/\Delta 17Ct}$, *Ankrd31*$^{-/-}$ and their respective wild-type control mice. Signal is represented as box plots (as defined in Methods) computed for hotspots (n= 13562) ranked according to increased DMC1-SSDS signal intensity and grouped into 10 bins (1 bin = 10% of the total hotspot number; bin 1 = weakest signal, bin 10= strongest signal).

c. Control for the sub-telomeric effect. Quantification was done as in Fig. 6f, but with ratios calculated between two random pools of 10 non-sub-telomeric consecutive hotspots (box plot as defined in Methods).

d. Sub-telomeric/Non-sub-telomeric hotspot density ratios for unchanged, decreased, and increased hotspots, as defined by DESeq2 analysis, for sub-telomeric regions defined using various distances from the telomeric annotated ends (1-2-3-4-5-7-10Mb). The log2 ratio was plotted and showed excess (positive values) or lack (negative values) of hotspots at sub-telomeric regions compared with non-sub-telomeric regions.

e. Distribution of hotspots within 3 Mb from annotated telomeric ends and according to their DESeq2 category (unchanged, decreased, or low signal). Note the absence of increased hotspots within this sub-telomeric window size. The normalized wild-type DMC1-SSDS signal is shown on the y-axis.

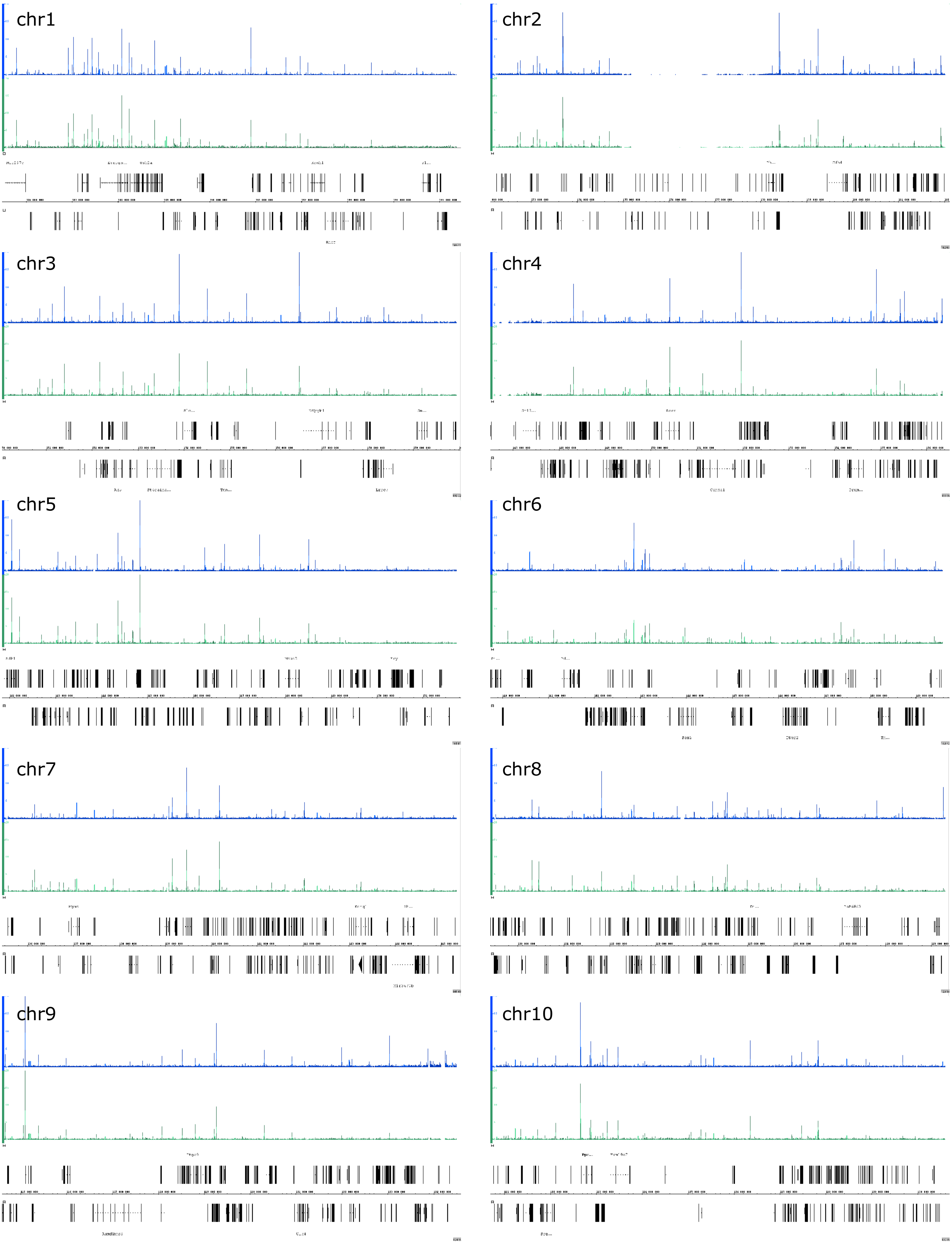

Supplementary Figure 14. Browser windows of DMC1-SSDS

Browser windows of DMC1-SSDS in wild-type (blue) and *Top6bl*$^{\Delta 17Ct/\Delta 17Ct}$ (green) mice within the 10Mb q-arm telomere-proximal regions of chromosomes 1 to 10.

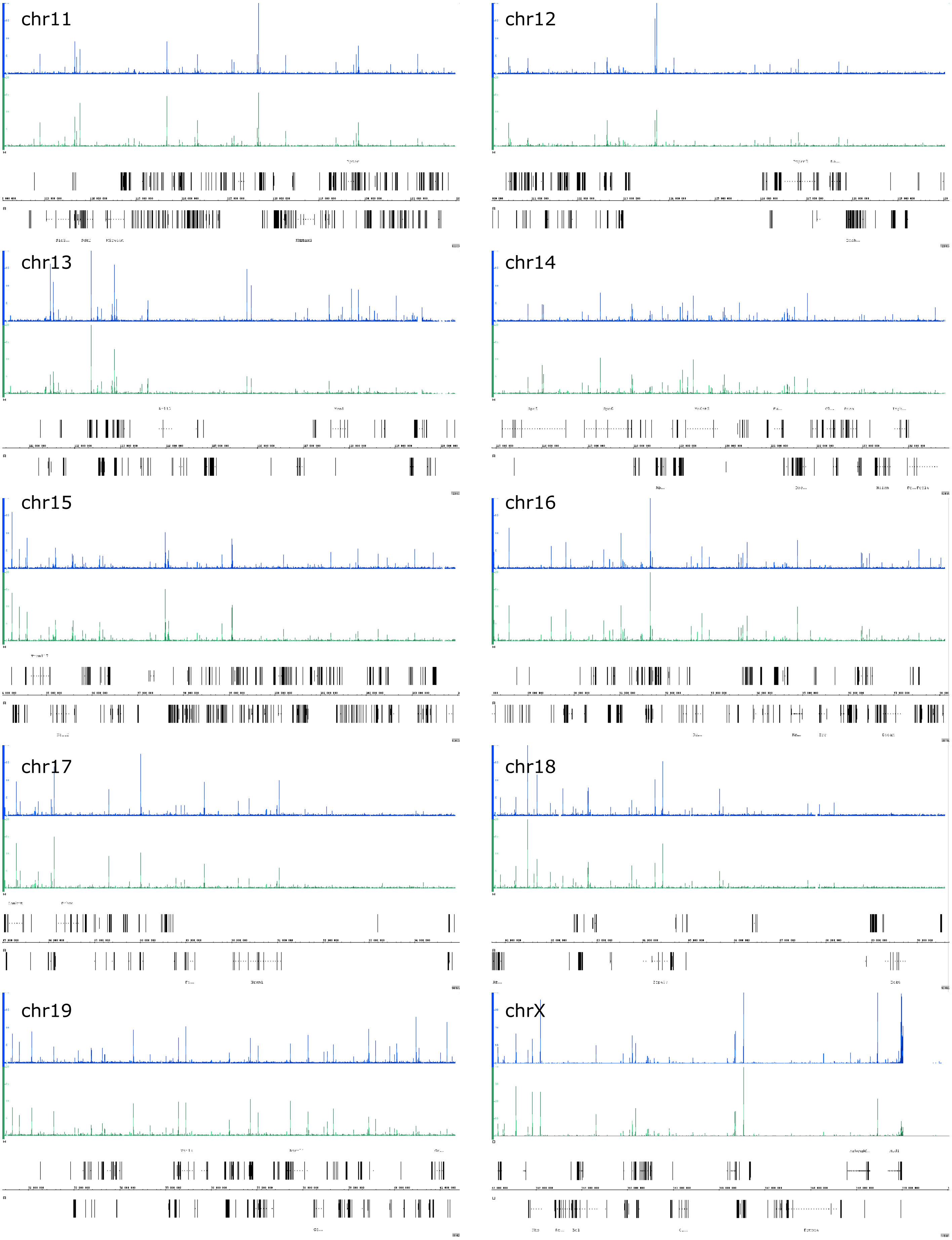

Supplementary Figure 15. Browser windows of DMC1-SSDS

Browser windows of DMC1-SSDS in wild-type (blue) and *Top6bl*$^{\Delta 17Ct/\Delta 17Ct}$ (green) mice within the 10Mb q-arm telomere-proximal regions of chromosomes 11 to X.

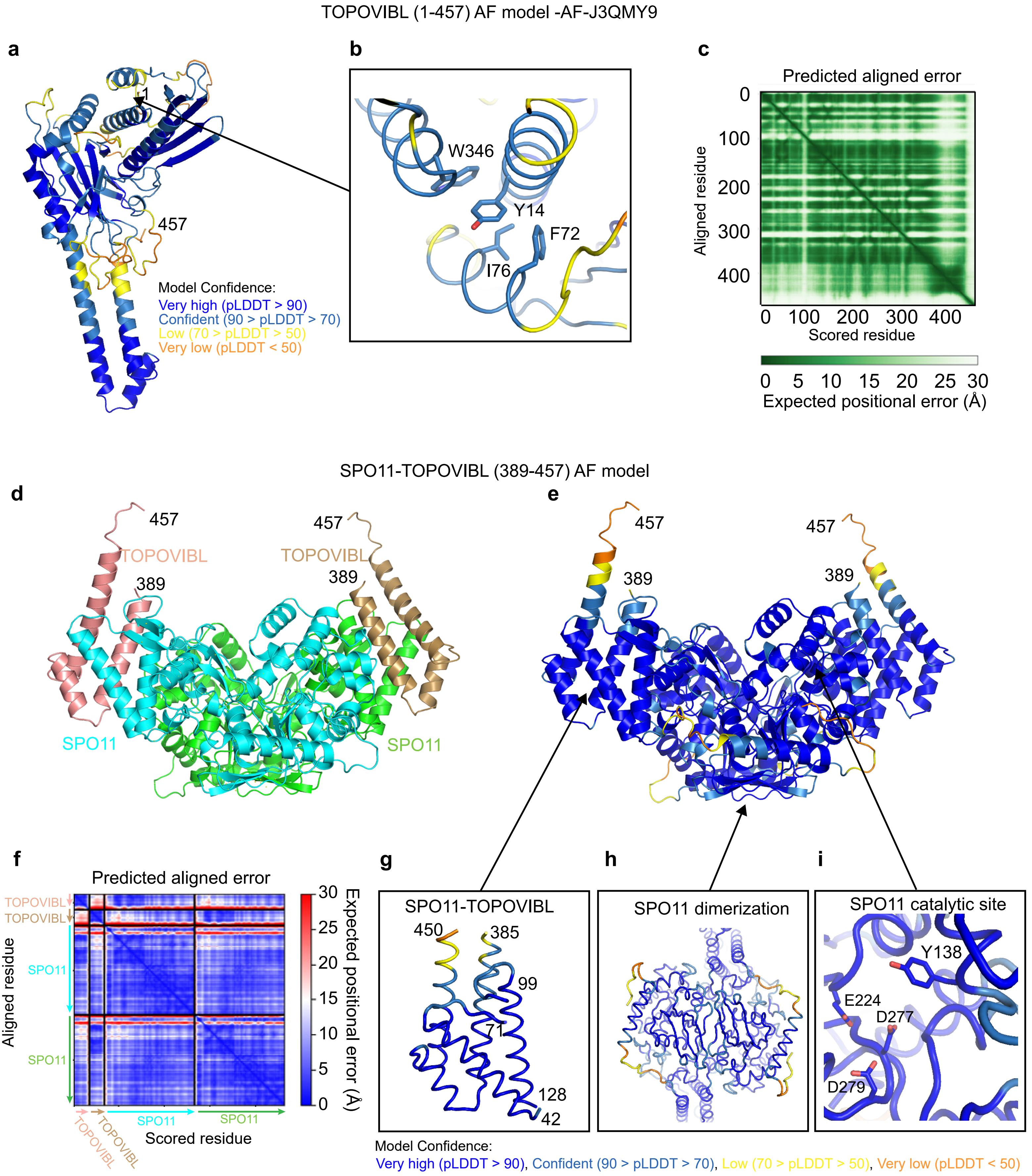

TOPOVIBL (1-457) AF model -AF-J3QMY9
a
1
457
Model Confidence:
Very high (pLDDT > 90)
Confident (90 > pLDDT > 70)
Low (70 > pLDDT > 50)
Very low (pLDDT < 50)
b
W346
Y14
F72
I76
c
Predicted aligned error
0
100
200
300
400
Aligned residue
0 100 200 300 400
Scored residue
0 5 10 15 20 25 30
Expected positional error (Å)
SPO11-TOPOVIBL (389-457) AF model
d
457
TOPOVIBL
389
457
TOPOVIBL
389
SPO11
SPO11
e
457
389
457
389
f
Predicted aligned error
TOPOVIBL
TOPOVIBL
SPO11
SPO11
Aligned residue
30
25
20
15
10
5
0
Expected positional error (Å)
TOPOVIBL
TOPOVIBL
SPO11
SPO11
Scored residue
g
SPO11-TOPOVIBL
450
385
99
71
128
42
h
SPO11 dimerization
i
SPO11 catalytic site
Y138
E224
D277
D279
Model Confidence:
Very high (pLDDT > 90), Confident (90 > pLDDT > 70), Low (70 > pLDDT > 50), Very low (pLDDT < 50)

Supplementary Figure 16. Quantitative evaluations of AlphaFold2 models

a. AlphaFold2 model of the mouse TOPOVIBL$^{1-457}$, including the GHKL-like, linker and transducer domains coloured according to the AlphaFold2 per-residue estimate of confidence (pLDDT).

b. Detail of the TOPOVIBL GHKL-like domain shown in Fig. 8b and coloured according to pLDDT.

c. Predicted aligned error plot for the model shown in **a**.

d. AlphaFold2 model of the SPO11-TOPOVIBL$^{389-457}$ complex coloured by protein chain.

e. AlphaFold2 model of the SPO11-TOPOVIBL$^{389-457}$ complex coloured according to pLDDT.

f. Predicted aligned error plot for the model shown in **d**.

g-i Details of the SPO11-TOPOVIBL complex shown in Fig. 8c-e coloured according to pLDDT.

	TOPVIBL-REC114
**Data collection**	
Space group	$P6_122$
Cell dimensions	
a, b, c (Å)	108.7, 108.7, 83
α β γ (°)	90, 90, 120
Resolution (Å)	62.3-2.26 (2.35-2.26)[a]
R_{merge} (%)	6.7(169)
I / σI	23.9 (1.4)
$CC_{½}$ (%)	99.9 (57.8)
Completeness (%)	84.4 (38.9)
Redundancy	18 (12.2)
**Refinement**	
Resolution (Å)	94.03-2.26
No. reflections	11285
R_{work} /R_{free}	0.226/0.249
B-factors	70
R.m.s. deviations	
Bond lengths (Å)	0.006
Bond angles (°)	1.412

[a] Values in parentheses are for highest resolution shell.

Supplementary Table 1

Data collection and refinement statistics.

	Unchanged-Decreased-Increased									
	Subtel 1M	Subtel 2M	Subtel 3M	Subtel 4M	Subtel 5M	Subtel 1M	Subtel 2M	Subtel 3M	Subtel 4M	Subtel 5M
chr1	0.19688	1	1	1	1	0.337954	0.707152	0.95226	1	0.30121
chr2	0.19688	1	0.9538	1	1	0.118562	0.728138	0.170249	0.26614	0.26614
chr3	NA	0.09331	0.00059	0	0.00062	NA	0.209354	0.000178	1.00E-06	8.70E-05
chr4	2.00E-05	0	0	0	0	1.20E-05	0	0	0	0
chr5	NA	0.09472	1	1	1	NA	0.214951	0.530377	1	1
chr6	0.1378	0.00765	1	1	1	0.275433	0.00955	0.263484	0.426418	0.848232
chr7	NA	1	1	1	1	NA	0.867484	1	1	0.92624
chr8	0.00204	9.00E-05	0.00548	0.2626	1	0.005766	5.50E-05	0.001712	0.039004	0.37051
chr9	2.00E-05	0	0	0	0	5.00E-06	0	0	0	0
chr10	0.07514	0.00201	0.06632	0.68638	1	0.213463	0.003835	0.016055	0.106033	0.211257
chr11	0.00041	0.00046	0.13714	0.58528	1	0.000331	0.000331	0.022116	0.06639	0.301525
chr12	NA	1	1	1	1	NA	1	1	0.877914	0.708196
chr13	NA	0.00056	0	0	0	NA	0.000478	0	0	0
chr14	0.00156	0.20581	0.25	1	1	0.004174	0.094238	0.094238	0.886754	0.784146
chr15	0.00041	0.58244	0.25	1	1	0.001482	0.189291	0.0572	0.170896	0.329774
chr16	0.85779	1	1	1	1	0.596137	1	1	0.932596	1
chr17	NA	NA	NA	1	0.02142	NA	NA	NA	0.622188	0.003701
chr18	NA	NA	1	1	1	NA	NA	1	0.644324	0.875619
chr19	0	0	0.00162	0.05963	0.26777	0	0	0.00019	0.004521	0.020866
chrX	NA	0	0	0	0	NA	0	0	0	0

Supplementary Table 2.

Statistics to test the biased distribution of hotspot densities at sub-telomeric vs. non-sub-telomeric regions in *Top6bl* $^{\Delta 17Ct}$ samples: Pearson's Chi square adjusted p-value for tests considering two (unchanged/decreased) or three (unchanged/decreased/increased) hotspot categories for different sizes of sub-telomeric regions. NA: not applicable due to the absence of hotspot with quantifiable signal.

Guides for CirspR	6509_Top6bl_sgRNA1	CCCGCAGGACTTGTGGCTAC
	6510_Top6bl_sgRNA2	GCAGGACTTGTGGCTACAGG
	6511_Top6bl_sgRNA3	TCCTGTAGCCACAAGTCCTG
Donor for CrispR	6512_Top6bl_donor1	TTCCTTGGCTTGCTGGGCGTCTCAGAGAGAATGCTTAGGACCTGTGTCCCGGATTCAGCCACTCGGACAGATTGGATACCTCCTGCAGCGCCAAGTCCTGCGGGAGCAGAGGAGGCGGTGGTGAGGA
	6513_Top6bl_donor2	GCGGCCTGGAGGTACCGCAGCGAGAGCAGGGCGGGTGCGGGCCTGAGCTACCACCGCCGGGTCCTCACCACCGCCTCCTCTGCTCCCGCAGGACTTGGCGCTGCAGGAGGTATCCAATCTGTCCGAG
Genoptyping	oliAN 320	CAACCATGGCAGGGAAGAATC
	oliAN 3201	TACCGACAGTAAACGCAGCC
RT-PCR *Top6bl*	Oli63	GGACCAGCTCTGCTACTTTT
	Oli70	CAGAGAGAATGCTTAGGACC
RT-PCR *Spo11*	Spo11:116U22	CTGTTGGCCATGGTGAAGAGAG
	Spo11:655L22	TGTGCCGACTAACATTCAAGGA

Supplementary Table 3

Oligonucleotides

Genotype	Pachytene with full autosomal synapsis (%)	Nuclei (n)
+/+	**76.6**	**77**
+/*$Top6bl^{W562A}$*	**86.7**	**30**
wild-type	**83.2**	**107**
**$Top6bl^{W562A}$/*$Top6bl^{W562A}$***	**86.4**	**81**
**$Top6bl^{W562A}$/*$Top6bl^{W562A}$***	**96.3**	**27**
**$Top6bl^{W562A}$ mutant**	**88.9**	**108**
+/+	**91.0**	**79**
+/*$Top6bl^{\Delta 17Ct}$*	**72.2**	**54**
wild-type	**88**	**133**
$Top6bl^{\Delta 17Ct}$/$Top6bl^{\Delta 17Ct}$	**84.4**	**96**
$Top6bl^{\Delta 17Ct}$/$Top6bl^{\Delta 17Ct}$	**85.2**	**108**
**$Top6bl^{\Delta 17Ct}$ mutant**	**85**	**204**

Supplementary Table 4

Percentage of pachytene spermatocytes with full synapsis
