# Supplementary material for: TOPOVIBL-REC114 interaction regulates meiotic DNA double-strand breaks
Source: Nat Commun. 2022 Nov 17;13:7048. doi: 10.1038/s41467-022-34799-0 (PMC9671922; doi:10.1038/s41467-022-34799-0)
Supplement: Supplementary file 3 — Reporting Summary [file 41467_2022_34799_MOESM3_ESM.pdf]

nature portfolio

Corresponding author(s): Dr Bernard de Massy (bernard.de-massy@igh.cnrs.fr) (CNRS Univ Montpellier)

Last updated by author(s): 01.11.2022

# Reporting Summary

Nature Portfolio wishes to improve the reproducibility of the work that we publish. This form provides structure for consistency and transparency in reporting. For further information on Nature Portfolio policies, see our Editorial Policies and the Editorial Policy Checklist.

## Statistics

For all statistical analyses, confirm that the following items are present in the figure legend, table legend, main text, or Methods section.

| n/a | Confirmed | |
|---|---|---|
| ☐ | ☒ | The exact sample size (*n*) for each experimental group/condition, given as a discrete number and unit of measurement |
| ☐ | ☒ | A statement on whether measurements were taken from distinct samples or whether the same sample was measured repeatedly |
| ☐ | ☒ | The statistical test(s) used AND whether they are one- or two-sided<br>*Only common tests should be described solely by name; describe more complex techniques in the Methods section.* |
| ☒ | ☐ | A description of all covariates tested |
| ☒ | ☐ | A description of any assumptions or corrections, such as tests of normality and adjustment for multiple comparisons |
| ☐ | ☒ | A full description of the statistical parameters including central tendency (e.g. means) or other basic estimates (e.g. regression coefficient) AND variation (e.g. standard deviation) or associated estimates of uncertainty (e.g. confidence intervals) |
| ☐ | ☒ | For null hypothesis testing, the test statistic (e.g. *F*, *t*, *r*) with confidence intervals, effect sizes, degrees of freedom and *P* value noted<br>*Give P values as exact values whenever suitable.* |
| ☒ | ☐ | For Bayesian analysis, information on the choice of priors and Markov chain Monte Carlo settings |
| ☒ | ☐ | For hierarchical and complex designs, identification of the appropriate level for tests and full reporting of outcomes |
| ☒ | ☐ | Estimates of effect sizes (e.g. Cohen's *d*, Pearson's *r*), indicating how they were calculated |

*Our web collection on statistics for biologists contains articles on many of the points above.*

## Software and code

Policy information about availability of computer code

| | |
|---|---|
| Data collection | Data was collected on the ESRF beamline ID30B using the MXcuBE3 software (ESRF). |
| Data analysis | The data were processed using autoPROC (55). Phases were obtained by molecular replacement using PHASER (56) . For crystallographic data analysis the following software was used: AutoProc, version 1.1.7; Phaser, version 2.8.2; Resolve, version 2.13; Coot, version 0.9.6; Refmac5, version 5.8.0267, Molprobity 4.5.1.<br>ITC data were analysed using the Origin software, version 7.0 (MicroCal). |

For manuscripts utilizing custom algorithms or software that are central to the research but not yet described in published literature, software must be made available to editors and reviewers. We strongly encourage code deposition in a community repository (e.g. GitHub). See the Nature Portfolio guidelines for submitting code & software for further information.

## Data

Policy information about availability of data

All manuscripts must include a data availability statement. This statement should provide the following information, where applicable:

- Accession codes, unique identifiers, or web links for publicly available datasets
- A description of any restrictions on data availability
- For clinical datasets or third party data, please ensure that the statement adheres to our policy

Data availibility
The DMC1-SSDS raw and processed data for this study have been deposited in the European Nucleotide Archive (ENA) at EMBL-EBI (accession number PRJEB43730) (https://www.ebi.ac.uk/ena/browser/view/PRJEB43730). Data are available upon request during the reviewing process. The atomic coordinates and structure factors of the mouse REC114-TOPOVIBL complex determined in this study have been deposited at the Protein Data Bank (http://www.ebi.ac.uk/pdbe/) under the PDB accession code 7QWV. Source data are provided with this paper

Data availibility
The DMC1-SSDS raw and processed data for this study have been deposited in the European Nucleotide Archive (ENA) at EMBL-EBI (accession number PRJEB43730) (https://www.ebi.ac.uk/ena/browser/view/PRJEB43730). Data are available upon request during the reviewing process. The atomic coordinates and structure factors of the mouse REC114-TOPOVIBL complex determined in this study have been deposited at the Protein Data Bank (http://www.ebi.ac.uk/pdbe/) under the PDB accession code 7QWV. Source data are provided with this paper

## Human research participants

Policy information about studies involving human research participants and Sex and Gender in Research.

| | |
|---|---|
| Reporting on sex and gender | *Use the terms sex (biological attribute) and gender (shaped by social and cultural circumstances) carefully in order to avoid confusing both terms. Indicate if findings apply to only one sex or gender; describe whether sex and gender were considered in study design whether sex and/or gender was determined based on self-reporting or assigned and methods used. Provide in the source data disaggregated sex and gender data where this information has been collected, and consent has been obtained for sharing of individual-level data; provide overall numbers in this Reporting Summary. Please state if this information has not been collected. Report sex- and gender-based analyses where performed, justify reasons for lack of sex- and gender-based analysis.* |
| Population characteristics | *Describe the covariate-relevant population characteristics of the human research participants (e.g. age, genotypic information, past and current diagnosis and treatment categories). If you filled out the behavioural & social sciences study design questions and have nothing to add here, write "See above."* |
| Recruitment | *Describe how participants were recruited. Outline any potential self-selection bias or other biases that may be present and how these are likely to impact results.* |
| Ethics oversight | *Identify the organization(s) that approved the study protocol.* |

Note that full information on the approval of the study protocol must also be provided in the manuscript.

# Field-specific reporting

Please select the one below that is the best fit for your research. If you are not sure, read the appropriate sections before making your selection.

☒ Life sciences ☐ Behavioural & social sciences ☐ Ecological, evolutionary & environmental sciences

For a reference copy of the document with all sections, see nature.com/documents/nr-reporting-summary-flat.pdf

# Life sciences study design

All studies must disclose on these points even when the disclosure is negative.

| | |
|---|---|
| Sample size | Sample size for follicles count were at least 10 sections, obtained from a minimum of 2 different mice per genotype. For fertility test, a minimum of four females were mated with males and sperm count were obtained from a number of animal ranging from 7 to 1 per genotype. For cytological analysis, a minimum of 30 nuclei at each meiosis prophase I substage were used to allow for statistical testing. Results were compared using the two-tailed non parametric Mann-Whitney test. |
| Data exclusions | No data were excluded |
| Replication | Cytological analysis were performed using at least two independent mice, for each genotype, and the result obtained reproduced.<br>The biochemical experiments were replicated using independent protein preparations. The cytological experiments were reproduced with independent mice, the IP were reproduced with independent protein extracts. All replicates were succesful. Protein expression and |

| | |
|---|---|
| | immunoprecipitation experiments were performed in triplicate, using three independent mice protein Information about replicates is provided in the section Methods , Statistics and Reproducibility |
| Randomization | For all experiments, each animals were chosen randomly among progenies of interest : Progenies were genotyped and genotypes of interest were randomly selected. Replicate were chosen from independent litters. |
| Blinding | For image analysis, foci counts and intensity are monitored by the image software, thus user independent. For the specific manual counting of bivalents (data from Fig. 7), AN generated the data, and images were analyzed blindly by either TR and BdM, and compared to the results obtained by AN. Results were found to be consistant. |

# Reporting for specific materials, systems and methods

We require information from authors about some types of materials, experimental systems and methods used in many studies. Here, indicate whether each material, system or method listed is relevant to your study. If you are not sure if a list item applies to your research, read the appropriate section before selecting a response.

## Materials & experimental systems

| n/a | Involved in the study | |
|---|---|---|
| ☐ | ☒ | Antibodies |
| ☒ | ☐ | Eukaryotic cell lines |
| ☒ | ☐ | Palaeontology and archaeology |
| ☐ | ☒ | Animals and other organisms |
| ☒ | ☐ | Clinical data |
| ☒ | ☐ | Dual use research of concern |

## Methods

| n/a | Involved in the study | |
|---|---|---|
| ☐ | ☒ | ChIP-seq |
| ☒ | ☐ | Flow cytometry |
| ☒ | ☐ | MRI-based neuroimaging |

## Antibodies

| | |
|---|---|
| Antibodies used | The following antibodies were used in the study with information about supplier, reference, dilution:<br>Rabbit polyclonal anti-GAD (1:3000; UPSTATE-Millipore 06-283), Rabbit polyclonal anti-GBD (1:1000; SIGMA; G3042).<br>Rabbit polyclonal anti-SYCP1 (1:400; Abcam, ab15090), rabbit polyclonal anti-DMC1 (1:200; Santa Cruz, H100), rabbit monoclonal anti-RPA2 (1:200; Abcam, ab76420 clone name EPR2877Y), mouse monoclonal anti-phospho-histone H2AX (Ser139) (γH2AX) (1:10000; Millipore, 05-636, clone name JBW301). The anti-REC114 is a rabbit monoclonal homemade antibody by Scott Keeney lab. Original commercial source cannot be specified, for further details see validation. Anti-rabbit LC mouse monoclonal secondary antibody (1:3000, Jackson ImmunoResearch 211-032-171, AB_2339149). |
| Validation | For anti-TOPOVIBL, rabbits were injected with full-length mouse His-TOPOVIBL protein prepared from E. coli inclusion bodies. Rabbit serum was purified by affinity using His-TOPOVIBL purified from inclusion bodies. Antibody specificity and affinity was verified on protein extracts obtained from mouse WT and Topo6blKO, and with in vitro translated TOPOVIBL protein. The following primary antibodies have been validated by the manufacturers: Rabbit polyclonal anti-SYCP1 (1:400; Abcam, ab15090) https://www.abcam.com/scp1-antibody; Rabbit monoclonal anti-RPA2 (1:200; Abcam, ab76420 clone name EPR2877Y), https://www.abcam.com/rpa32rpa2-antibody-epr2877y-ab76420.html; Mouse monoclonal anti-phospho-histone H2AX (Ser139) (γH2AX) (1:10000; Millipore, 05-636, clone name JBW301) https://www.merckmillipore.com/FR/fr/product/Anti-phospho-Histone-H2A.X-Ser139-Antibody-clone-JBW301,MM_NF-05-636?ReferrerURL=https%3A%2F%2Fwww.google.com%2F#. The following non-commercial antibodies have been validated in the corresponding studies:<br>anti-SYCP3: Grey, C. et al. PLoS Biol 7, e35 (2009).<br>anti-MEI4: Kumar, R. et al. Genes Dev 24, 1266-80 (2010).<br>anti-REC114: Kumar, R. et al. Life Sci Alliance 1, e201800259 (2018).<br>anti-ANKRD31: Papanikos, F. et al. Mol Cell 74, 1069-1085 e11 (2019).<br>anti-IHO1: Stanzione, M. et al. Nat Cell Biol 18, 1208-1220 (2016).<br>anti-REC114: Acquaviva, L. et al. Nature 582, 426-431 (2020). |

## Animals and other research organisms

Policy information about [studies involving animals](); [ARRIVE guidelines]() recommended for reporting animal research, and [Sex and Gender in Research]()

| | |
|---|---|
| Laboratory animals | Mice Mus Musculus domesticus males at 12, 14dpp and embryonic females at 15, 16 and 17 dpc were used for this study. Adult male and females mice from 2.3 to 10.6 months were used. Mouse strains: Mice were in the C57BL/6J background. Mice carrying the homozygous mutant alleles Top6bl<em1(W562A)BdM> and Top6bl<em2(delta17)BdM> were named Top6blW562A/W562A and Top6bl 17Ct/ 17Ct, respectively. Top6bl-/- mice carry the Gm960em2Arte allele, a null allele due to a 5bp deletion in Top6bl (5). Standard conditions were use for animal housing. |
| Wild animals | The study did not involve wild animals. |

Reporting on sex | This study reports phenotypic analysis of both male and female mice.

Field-collected samples | The study did not involve samples collected from the field.

Ethics oversight | All experiments were carried out according to the CNRS guidelines and were approved by the ethics committee on live animals (project CE-LR-0812 and 1295). The regional ethic committee is registered to the Comité National de Réflexion Ethique sur l'Expérimentation Animale .

Note that full information on the approval of the study protocol must also be provided in the manuscript.

# ChIP-seq

## Data deposition

☒ Confirm that both raw and final processed data have been deposited in a public database such as GEO.

☒ Confirm that you have deposited or provided access to graph files (e.g. BED files) for the called peaks.

Data access links
*May remain private before publication.*

https://www.ebi.ac.uk/ena/browser/view/PRJEB43730

Files in database submission

17144FL-05-01-02_S2_L001_R2_001.fastq.gz ; 17144FL-05-01-02_S2_L001_R1_001.fastq.gz ;
17144FL-05-02-02_S4_L002_R2_001.fastq.gz ; 17144FL-05-02-02_S4_L002_R1_001.fastq.gz ;
17144FL-05-02-01_S3_L002_R2_001.fastq.gz ; 17144FL-05-02-01_S3_L002_R1_001.fastq.gz ;
17144FL-05-01-01_S1_L001_R2_001.fastq.gz ; 17144FL-05-01-01_S1_L001_R1_001.fastq.gz ;
CHIP_DMC1_Top6blDelta17Ct_Rep0_nochrY.tab ; CHIP_DMC1_Top6blWT_Rep0_nochrY.tab ;
17144FL-05-01-02_S2_L001_trim_PF_bwara_mm10_noPARy_UNIQUE_SORT_DEDUP_SORT.ssDNA_type1.bed.gz ;
17144FL-05-02-02_S4_L002_trim_PF_bwara_mm10_noPARy_UNIQUE_SORT_DEDUP_SORT.ssDNA_type1.bed.gz ;
17144FL-05-02-01_S3_L002_trim_PF_bwara_mm10_noPARy_UNIQUE_SORT_DEDUP_SORT.ssDNA_type1.bed.gz ;
17144FL-05-01-01_S1_L001_trim_PF_bwara_mm10_noPARy_UNIQUE_SORT_DEDUP_SORT.ssDNA_type1.bed.gz ;
17144FL-05-01-02_S2_L001_trim_PF_bwara_mm10_noPARy_UNIQUE_SORT_DEDUP_SORT.bam ;
17144FL-05-02-02_S4_L002_trim_PF_bwara_mm10_noPARy_UNIQUE_SORT_DEDUP_SORT.bam ;
17144FL-05-02-01_S3_L002_trim_PF_bwara_mm10_noPARy_UNIQUE_SORT_DEDUP_SORT.bam ;
17144FL-05-01-01_S1_L001_trim_PF_bwara_mm10_noPARy_UNIQUE_SORT_DEDUP_SORT.bam

Genome browser session
(e.g. UCSC)

no longer applicable

## Methodology

Replicates | 2 replicates for the mutant mice and 2 replicates for the control mice.

Sequencing depth | Paired-end Illumina sequencing: 2x150bp
Sequencing depth: 45-55Mo of read fragments

Antibodies | goat anti-DMC1 antibody (Santa Cruz, C-20)

Peak calling parameters

After quality control and read trimming to remove adapter sequences and low-quality reads, DMCl ChlP SSDS reads were mapped to the UCSC mouse genome assembly build GRCm38/mml0. The previously published method was used for DMCl-SSDS read mapping (i.e. the BWA modified algorithm and their customized script that were specifically developed to align and recover ssDNA fragments). A filtering step was performed on the aligned reads to keep only non-duplicated and high-quality uniquely mapped reads with no more than one mismatch per read. To identify meiotic hotspots from biologically replicated samples in DMCl-SSDS, the lrreproducible Discovery Rate (IDR) method was used, as done in our previous studies. This method was developed for ChlP-seq analysis and extensively used by the ENCODE and modENCODE projects. The framework developed by Qunhua Li and Peter Bickel's group (https://sites.google.com/site/anshulkundaje/projects/idr) was followed. Briefly, this method allows testing the reproducibility within and between replicates by using IDR statistics. Following their pipeline, peakcalling was performed using MACS version 2.0.10 with relaxed conditions (--pvalue=0. l --bwlO00 --nomodel --shift400) for each of the two replicates, the pooled dataset, and pseudoreplicates
that were artificially generated by randomly sampling half of the reads twice for each replicate and the pooled dataset.
Then IDR analyses were performed and reproducibility was checked. Final peak sets were built by selecting the top N peaks from pooled datasets (ranked by increasing p values), with N defined as the highest value between Nl (the number of overlapping peaks with an IDR below 0.01, when comparing pseudo-replicates from pooled datasets) and N2 (the number of overlapping peaks with an IDR below 0.05 when comparing the true replicates, as recommended for the mouse genome). Hotspot centring and strength calculation were computed following the method described by Khil et al. Ali read distributions and signal intensities presented in this work were calculated after pooling reads from both replicates, if not otherwise stated. When DSB maps were compared between mouse genotypes, the lbp-overlaps were restricted to the central 400bp of hotspots (+/- 200bp around the peak centre). For correlation plots, the type 1 single-strand DNA signal was library-normalized (fragment per million)

Data quality | We used the IDR methodology to recover high quality and reproducible peaks.

Software | We used publicly available software and scripts to recover peaks and analyse them, and mentioned them in the Methods section providing the proper references. For DMC1-SSDS analysis, we used bwa (version 0.5.9rc1) and modified script of bwa (previously

published (Khil et al. 2012)), MACS (version 2.0.10) for peakcalling, IDR code (version 2012 available here https://sites.google.com/site/anshulkundaje/projects/idr/deprecated ), R (version 4.0.3), DESeq2 (version 1.30.1).
